# Gut Microbiota and Intestinal Monodomination as a Predictor for Bacteremia in Allogeneic Hematopoietic Cell Transplant Recipients

**DOI:** 10.1093/infdis/jiag005

**Published:** 2026-02-24

**Authors:** M M DeMeules, S C Proll, X Hua, S Srinivasan, T Loeffelholz, C Liu, M C Wu, T L Fiedler, N G Hoffman, L A Bourassa, S A Pergam, D N Fredricks

**Affiliations:** Vaccine and Infectious Disease Division, Fred Hutchinson Cancer Center, Seattle, Washington, USA; Vaccine and Infectious Disease Division, Fred Hutchinson Cancer Center, Seattle, Washington, USA; Public Health Sciences Division, Fred Hutchinson Cancer Center, Seattle, Washington, USA; Vaccine and Infectious Disease Division, Fred Hutchinson Cancer Center, Seattle, Washington, USA; Vaccine and Infectious Disease Division, Fred Hutchinson Cancer Center, Seattle, Washington, USA; Vaccine and Infectious Disease Division, Fred Hutchinson Cancer Center, Seattle, Washington, USA; Public Health Sciences Division, Fred Hutchinson Cancer Center, Seattle, Washington, USA; Vaccine and Infectious Disease Division, Fred Hutchinson Cancer Center, Seattle, Washington, USA; Department of Laboratory Medicine, University of Washington, Seattle, Washington, USA; Department of Laboratory Medicine and Pathology, University of Washington, Seattle, Washington, USA; Vaccine and Infectious Disease Division, Fred Hutchinson Cancer Center, Seattle, Washington, USA; Division of Allergy and Infectious Diseases, University of Washington, Seattle, Washington, USA; Vaccine and Infectious Disease Division, Fred Hutchinson Cancer Center, Seattle, Washington, USA; Division of Allergy and Infectious Diseases, University of Washington, Seattle, Washington, USA; Department of Medicine, University of Washington, Seattle, Washington, USA

**Keywords:** microbiota, bacteremia, hematopoietic cell transplant

## Abstract

**Background:**

Bacteremia is a frequent complication in patients undergoing allogeneic hematopoietic cell transplantation (HCT). Alterations to the gut microbiota after HCT have been associated with adverse outcomes including bacteremia and reduced overall survival. Previous studies suggest that loss of gut bacterial diversity and domination by a single species may predict bloodstream infections, but the degree of domination leading to the optimal positive predictive value (PPV) has not been defined.

**Methods:**

Stool samples were collected weekly from allogeneic HCT recipients and were analyzed by 16S rRNA gene PCR with sequencing to determine gut microbiota composition and document domination events. Bacteremia events were captured by review of medical records. The PPV for bacteremia of any detection of that species in stool and for domination events at 10%, 30%, and 50% abundance were calculated.

**Results:**

Of 277 HCT recipients, 95 experienced bacteremia, with 130 bacteremia events. Intestinal domination was associated with but not highly predictive for bacteremia, reflected by low PPV. Presence of coagulase-negative *Staphylococcus* in the gut at >30% relative abundance was associated with increased risk of coagulase-negative *Staphylococcus* bloodstream infections with PPV of 38%.

**Conclusions:**

Hematopoietic cell transplantation is associated with significant disruption to the gut microbiota, particularly in patients who subsequently develop bacteremia. Intestinal domination may not be as useful as previously thought given its low PPV for most species implicated in bloodstream infections. The association between gut colonization with *Staphylococcus* and bacteremia events suggests that the gut may be an under-recognized portal of entry for these organisms in patients after HCT.

## Teaser

Our article explores the association between bacteremia and gut microbial composition among HCT recipients. We assess the predictive ability of the gut microbiota, as well as the veracity of bacteremia events caused by coagulase-negative *Staphylococcus* species in this population.

Bacteremia is a common complication of allogeneic hematopoietic cell transplantation (HCT) used to treat hematologic malignancies and disorders [1]. Bacteremia episodes are linked to prolonged hospital stays and higher mortality [2]. Bacteria may enter the bloodstream from numerous sites, including the mouth or gut from mucosal damage, skin via venous catheters, and urinary tract or respiratory tract from infections. During HCT, the gastrointestinal mucosal barrier can become damaged due to chemo-radiation, allowing bacteria to enter the bloodstream, termed bacteremia due to mucosal barrier injury (MBI) [3].

Hematopoietic cell transplantation often results in a disturbed gut microbiota, manifested as loss of species diversity and domination by bacteria such as *Enterococcus* and Pseudomonadota (Proteobacteria) [4, 5]. Reduced diversity may lead to impaired colonization resistance against pathogens [6]. Intestinal monodomination, or domination with one species based on 16S rRNA sequencing of stool samples, is often associated with poor health outcomes [7, 8].

Previous studies have demonstrated a connection between bacterial species found in the gut and species implicated in bloodstream infections (BSI) of HCT recipients [9]. Tamburini et al performed phylogenetic analysis of gut bacterial sequences compared to BSI organisms in HCT recipients and found these bacterial strains were more closely related within patients than between patients, supporting the hypothesis that BSI isolates can arise from the patient's own gut microbiota [9]. The same conclusion was found by Kelly et al, who utilized prospective fecal sampling and comparative genomics to demonstrate that BSI isolates are most similar, and often identical, to fecal bacterial strains that are sampled immediately preceding BSI onset [10].

What remains unclear is the temporal association between intestinal colonization or domination and risk of bacteremia, and how best to define domination for the purpose of assessing bacteremia risk. One definition of intestinal domination in HCT recipients used a cutoff of >30% relative abundance and found the median time between intestinal monodomination and bacteremia was 7 days [7].

Our primary aim was to develop a definition of intestinal domination that is based on its predictive strength for bacteremia with the future goal of employing directed antibiotic therapy to reduce bacteremia risk and mortality. We performed PCR with 16S rRNA gene sequencing on weekly stool samples from HCT recipients beginning pre-transplant through 120 days post-transplant. We compared presence and abundance of bacteria in the gut (stool) to bacteria implicated in bacteremia episodes.

## METHODS AND MATERIALS

### Study Population

Three hundred and twenty-nine HCT recipients over age 18 were enrolled in a prospective study of the gut microbiota and outcomes at the Fred Hutchinson Cancer Center in Seattle, WA, from April 2014 to May 2019. Forty-seven patients were excluded as they never underwent transplant or had inadequate stool sample submission, and 5 patients were excluded due to bacteremia occurring outside the transplant window (Supplementary Figure 1). Remaining patients were divided into 2 groups: those who never experienced a bacteremia event and those who experienced a bacteremia event between day 0 and day 120 post-transplant (bacteremia) (Table 1). In another analysis, we analyzed stool samples from 50 healthy HCT donors. Written, informed consent was obtained before enrollment. This study was approved by the IRB at Fred Hutchinson Cancer Center. Levofloxacin neutropenic prophylaxis is the standard of care at this institution.

**Table 1.** HCT Patient Demographics and Transplant Characteristics

	All Patients (n = 277)	Non-bacteremia (n = 182)	Bacteremia (n = 95)
Age (years)			
Range	20–77	20–77	22–74
Average	54	54	51
Sex			
Male	170 (61%)	110 (60%)	60 (63%)
Female	107 (39%)	72 (40%)	35 (37%)
Race			
American Indian/Alaskan Native	1 (<1%)	1 (<1%)	0
Asian	16 (6%)	12 (7%)	4 (4%)
Black or African American	7 (3%)	2 (1%)	5 (5%)
Native Hawaiian or Pacific Islander	2 (<1%)	1 (<1%)	1 (1%)
White	207 (75%)	137 (75%)	70 (74%)
More than one race	16 (6%)	11 (6%)	5 (5%)
Unknown or not reported	28 (10%)	18 (10%)	10 (11%)
Donor match			
Match, related	66 (24%)	49 (27%)	17 (18%)
Mismatch, related	2 (<1%)	2 (1%)	0
Match, unrelated	149 (54%)	97 (53%)	52 (55%)
Mismatch, unrelated	22 (8%)	18 (10%)	4 (4%)
Haploidentical	14 (5%)	10 (5%)	4 (4%)
Cord blood	24 (9%)	6 (3%)	18 (19%)
Transplant type			
Bone marrow	16 (6%)	11 (6%)	5 (5%)
Cord	24 (9%)	6 (3%)	18 (19%)
PBSC^a^	237 (86%)	165 (91%)	72 (76%)
Underlying disease			
Aplastic anemia	7 (3%)	4 (2%)	3 (3%)
Acute lymphoblastic leukemia	26 (9%)	13 (7%)	13 (14%)
Acute myeloid leukemia	97 (35%)	64 (35%)	33 (35%)
Chronic lymphocytic leukemia	6 (2%)	4 (2%)	2 (2%)
Chronic myeloid leukemia	8 (3%)	6 (3%)	2 (2%)
Other leukemia^b^	13 (5%)	10 (5%)	3 (3%)
Lymphoma	24 (9%)	18 (10%)	6 (6%)
Multiple myeloma	8 (3%)	6 (3%)	2 (2%)
Myelodysplastic syndrome	10 (4%)	5 (3%)	5 (5%)
Myelofibrosis	30 (11%)	21 (12%)	9 (9%)
Refractory anemia	33 (12%)	20 (11%)	13 (14%)
Other^c^	15 (5%)	11 (6%)	4 (4%)

Abbreviation: PBSC, peripheral blood stem cells.

^a^One patient received both PBSC and bone marrow for transplant, which we coded as PBSC.

^b^“Other leukemia” includes acute myelomonocytic leukemia; acute monocytic leukemia; acute promyelocytic leukemia; chronic leukemia; chronic myelomonocytic leukemia; and leukemia (not otherwise specified).

^c^“Other” includes blastic plasmacytoid dendritic cell neoplasm; Crohn's disease; erythropoietic protoporphyria; hemophagocytic lymphohistiocytosis; mycosis fungoides; myeloproliferative disease; paroxysmal nocturnal hemoglobinuria; and refractory cytopenia with multilineage dysplasia.

### Sample Collection and Microbiota Characterization

Weekly stool swabs were processed following previously established protocols [11]. One stool swab was collected within 15 days pre-transplant and up to once per week for 120 days post-transplant. Swabs were stored at −80°C within 72 h of collection and remained frozen until processing and DNA extraction. Broad-range 16S rRNA gene PCR with sequencing was used to generate relative abundance data. Additional information on gut microbiota mock community and reference sets is found in Supplementary Table 1*A–D*.

See Supplementary Data for additional methods.

## RESULTS

### Study Population and Sample Collection

A total of 277 HCT patients contributed 2316 stool swabs, with an average of 8 samples per patient. There were 95 patients who experienced at least one positive blood culture during the first 120 days post-transplant and 85 patients submitted at least one stool swab within 30 days of positive blood culture onset (Supplementary Figure 1). For complete details on age, sex, race, donor-matching, and underlying disease, see Table 1.

### Bacteremia Events and Bacteremia Onset Relative to Transplant

**Figure 1.**
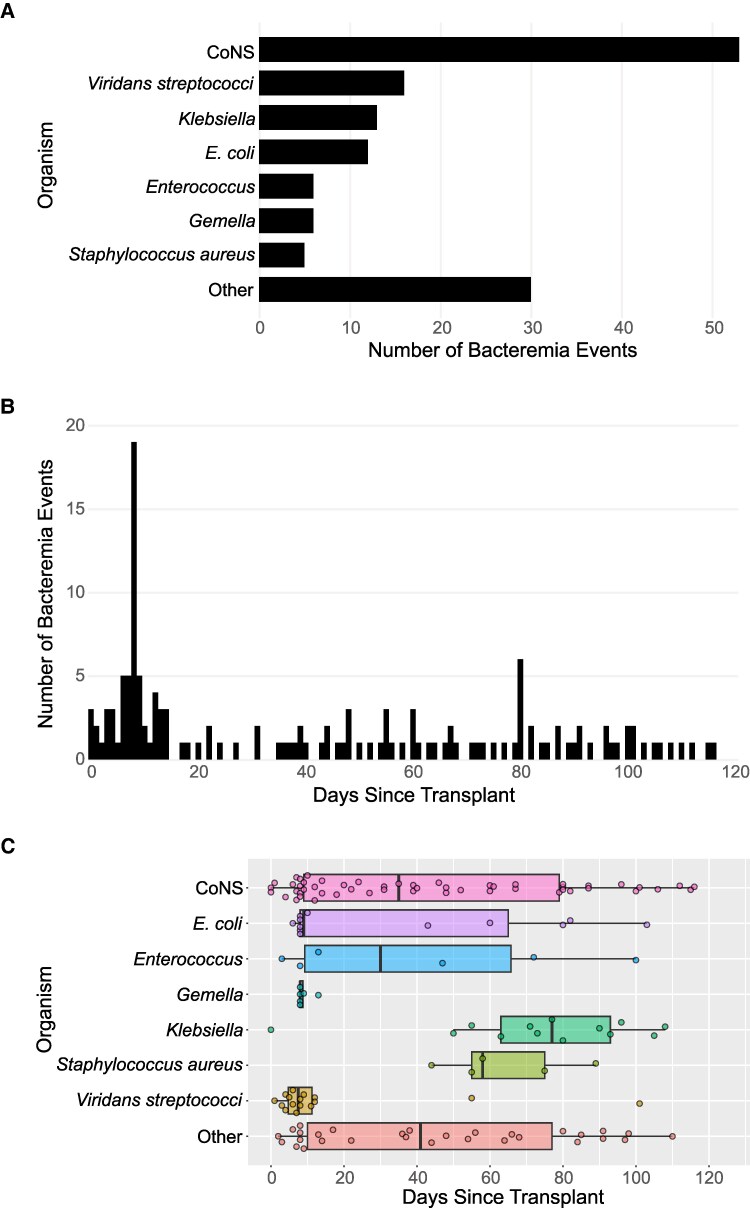
Bacteremia events by organism and day from transplant. *A*, The number of bacteremia events among the top 7 most common organisms implicated in bacteremia, along with the number of all other events caused by other organisms. *B*, Timeline of the post-transplant period depicting the number of bacteremia events on each day. *C*, The timeline of when each bacteremia event occurred for the top 7 organisms, along with all other organisms. There were 130 episodes of bacteremia among the 95 patients who experienced bacteremia post-transplant. Fifty-three (41%) bacteremia events occurred within the first 15 d post-transplant. For “Other” bacteria, please see Supplementary Figure 2.

There were 130 bacteremia events among the 95 patients in our cohort. Characteristics of bacteremia events are detailed in Supplementary Table 2. Most patients (n = 70) experienced one bacteremia event, while 16 experienced 2 events, and 9 experienced 3 or more events. Of the 130 bacteremia events, 14 (11%) involved multiple species. The most common organisms involved in bacteremia events were coagulase-negative *Staphylococcus* (CoNS) spp. (53 episodes), *Escherichia coli* (12), *Streptococcus mitis* group (11), and *Klebsiella pneumoniae* (10) (Figure 1). A complete list of organisms involved in each bacteremia event is in Supplementary Figure 2. Cord blood transplant (CBT) recipients had a higher rate of bacteremia, with 18/24 (75%) CBT recipients experiencing 21 bacteremia events (predominantly CoNS and *Viridans streptococci*). Similarly, HCT recipients with an underlying diagnosis of ALL had more bacteremia events, with 13/26 (50%) ALL patients experiencing 16 bacteremia events (also predominantly CoNS and *V. streptococci*).

Every bacteremia event was tracked relative to when transplant occurred. The median time to bacteremia was 14 days (interquartile range 8–65) after transplant, with the most bacteremia events on day 9. Figure 1 highlights an initial peak in the first 2 weeks followed by a more stable incidence between day 15 and day 100. Some organisms (*V. streptococci*, *Gemella*, and *E. coli*) were associated with bacteremia earlier in the post-transplant period, while other organisms (*Klebsiella* and *Staphylococcus aureus*) predominantly appeared in BSIs more than 40 days post-transplant.

### Microbiota Diversity Changes and Overall Survival in Patients With and Without Bacteremia

**Figure 2.**
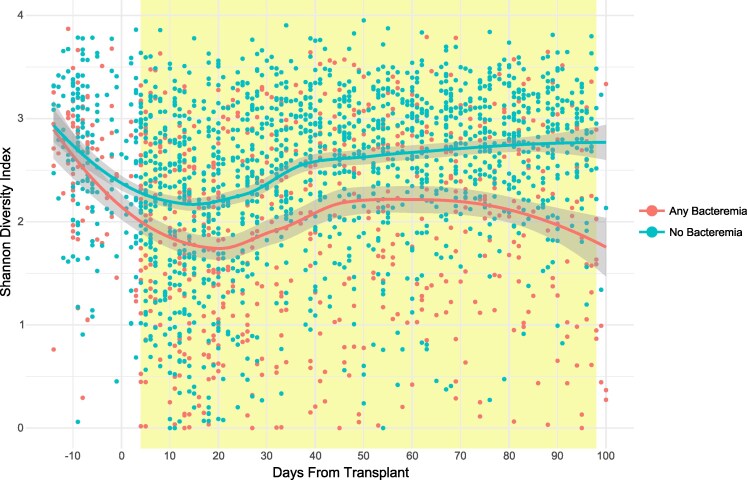
Changes in gut microbiota diversity during HCT. Gut bacterial diversity was measured from pre-transplant to 100 d post-transplant. Diversity is based on bacterial species detected in stool by 16S rRNA gene PCR with sequencing and calculated using Shannon Diversity Index (SDI). Regions highlighted in yellow indicate statistically significant differences (*P* < .05) between any bacteremia and no bacteremia groups.

We evaluated the change in gut microbiota diversity relative to transplant time (Figure 2). Patients experienced a decrease in gut bacterial diversity pre-transplant during conditioning. This decrease became more pronounced immediately after transplant. Gut diversity began increasing in most patients around day 20 post-transplant. Patients with bacteremia experienced a greater decrease in gut bacterial diversity post-transplant and did not recover microbial diversity to the same extent as patients without bacteremia, demonstrated in Figure 2 with bacteremia patients having a significantly less diverse gut microbiota compared to non-bacteremia patients throughout nearly the entire post-transplant period. These results did not change significantly when we excluded samples collected after bacteremia, at which time additional antibiotics were started (Supplementary Figure 3*A*). A similar pattern was seen when bacteremia patients were divided into “early” v “late” bacteremia, with patients who had early bacteremia events demonstrating a steeper decrease in microbial diversity initially, and late bacteremia patients demonstrating overall decreased diversity later in the post-transplant timeframe (Supplementary Figure 3*B*). Supplementary Figure 4 displays the relative abundance of bacterial genera from all stool samples collected by bacteremia and non-bacteremia patients.

We also explored differences in gut microbial composition within sub-groups of HCT recipients who had higher rates of bacteremia, specifically CBT recipients (Supplementary Table 3*A*) and patients with an underlying diagnosis of ALL (Supplementary Table 3*B*). Both CBT recipients and ALL patients demonstrated a higher abundance of gut microbial species that were associated with bacteremia events. Cord blood transplant patients also exhibited prolonged neutropenia post-transplant—an average of 24 days compared to 13 days for other types of transplants (*P*-value .002).

**Figure 3.**
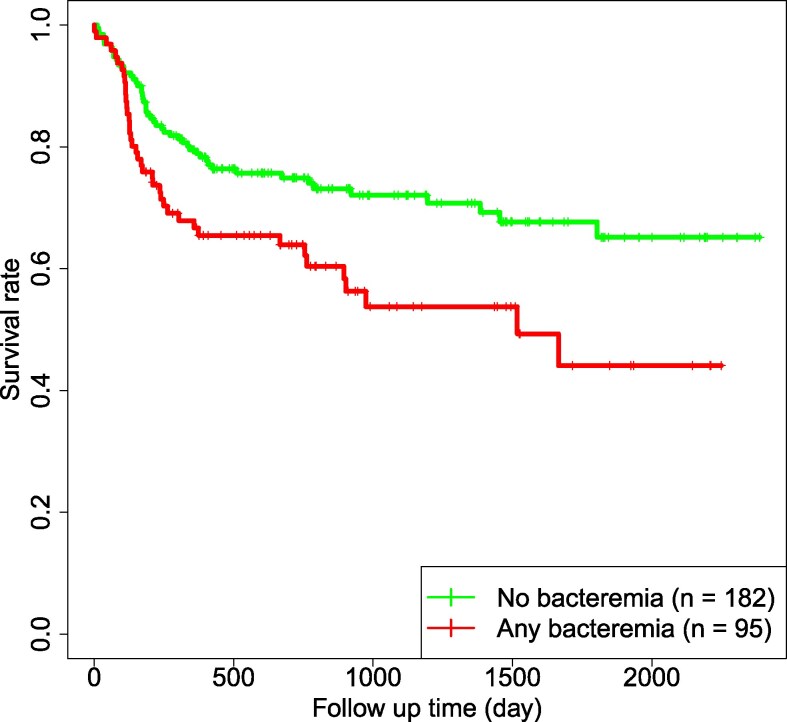
Association between bacteremia and mortality (*P* = .007).

Patient outcomes were followed for at least 1000 days post-transplant. Bacteremia was significantly associated with lower overall survival post-transplant (Figure 3).

### Gut Microbiota Genera Associated With Bacteremia and Concordance Between Blood and Stool Organisms

On examination of bacterial genera associated with bacteremia risk, we noted that coagulase-negative *Staphylococcus*, *Enterococcus*, and *Lactobacillus* were significantly associated with bacteremia (Supplementary Table 4, highlighted in blue), whereas *Roseburia*, *Blautia*, and *Anaerobutyricum* were negatively associated with bacteremia risk (Supplementary Table 4, highlighted in green). To evaluate potential seeding of the bloodstream by gut bacteria, we assessed how many bacteremia events were associated with presence of the same organism in the gut (Supplementary Table 5). Only patients who provided at least one stool swab within 30 days before or after bacteremia onset were included; 114 bacteremia episodes among 84 patients were analyzed. Of 114 bacteremia events, 67 (59%) were associated with presence of the bacteremia organism in the stool within 30 days of bacteremia. Thirty-four events had no species or genus match between the bloodstream organisms and stool organisms. The remaining 13 events were associated with a match at the genus level only.

### Positive Predictive Value of Bacteria in Stool for Predicting Bacteremia Events

**Figure 4.**
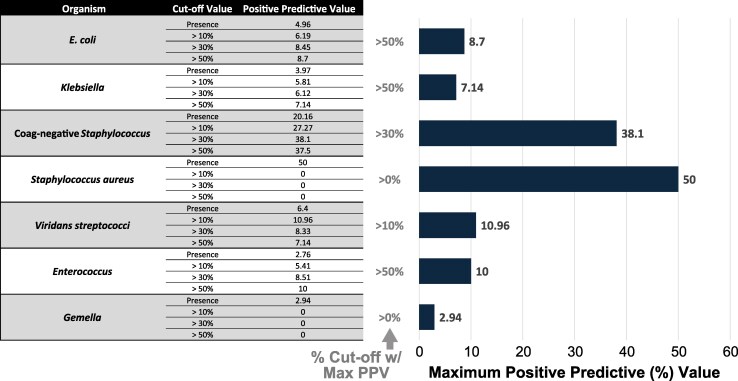
Positive predictive value for species. The presence and abundance of stool species were utilized to determine PPV for bacteremia events with different thresholds as noted in first 3 columns (left). The maximum PPV is displayed on the bar graph (right panel), corresponding to the threshold that represents the abundance level that achieves this PPV. Coagulase-negative *Staphylococcus* and *S. aureus* have the largest PPVs at 38.1% (for abundance > 30%) and 50% (for presence), respectively. Positive predictive value for other species is modest and ≤11%.

We evaluated the positive predictive value (PPV) of stool bacterial species for predicting bacteremia among the 7 most common organisms implicated in bacteremia events in our study. Positive predictive value was assessed for stool bacteria at various thresholds, including presence (any detection), >10%, >30%, and >50% relative abundance by 16S rRNA gene sequence reads. Most species had less than a 10% PPV at all cutoffs, except coagulase-negative *Staphylococcus*, at which a >30% relative abundance corresponds to a 38.1% PPV, and *S. aureus*, when presence of the organism in the stool corresponds to a 50% PPV, though with few events (Figure 4). A secondary analysis of PPV was performed excluding samples collected after bacteremia events, and our results remain largely unchanged (Supplementary Table 6, see highlighted values). Our primary analysis included samples within 30 days before or after bacteremia events to account for microbial changes that may be missed by only examining samples prior to bacteremia events and to reflect the imperfect nature of sampling in a clinical setting. Several additional analyses were performed to assess PPV within subpopulations, including those with gut GvHD later in the post-transplant period (Supplementary Table 7) and those with coagulase-negative *Staphylococcus* bacteremia events divided into early versus late bacteremia events (Supplementary Table 8).

### Coagulase-negative *Staphylococcus* Bacteremia Events and Gut Colonization

Of the 277 HCT recipients, 130 (46.9%) patients had CoNS detected in their stool. We further assessed the validity of CoNS detection in stool by defining CoNS colonization as CoNS at >1% relative abundance for at least 2 consecutive stool samples. Of the entire HCT recipient population with at least 2 stool samples (n = 262), 21 patients (8%) met this criterion (Table 2). Eight of 21 patients with CoNS gut colonization developed CoNS bacteremia (38.1%) compared to 34 of 241 patients (14.1%) who developed CoNS bacteremia without gut colonization (*P* = .004). None of the 50 healthy donors in our study had CoNS present in their stool samples.

**Table 2.** Coagulase-Negative *Staphylococcus* Bacteremia Events and Gut Colonization

	CoNS Colonization in Gut (n = 21)	No CoNS Colonization in Gut (n = 241)
Bacteremia	11 (52.4%)	79 (32.8%)
CoNS bacteremia	8 (38.1%)	34 (14.1%)

We examined the rate of bacteremia, both with CoNS and all other species, among patients who had CoNS colonization in the gut. Patients who had CoNS colonization in the gut were more likely to have bacteremia, either CoNS bacteremia or other, compared to patients without CoNS gut colonization.

We sought to determine if there was a difference in rate of detection of CoNS in stool samples from patients with CoNS bacteremia compared to patients with other bacteremia events and those without any bacteremia. The *prevalence* of bacteremia due to CoNS when patients had >30% relative abundance CoNS in stool was 5.6%, compared to 0.9% prevalence of bacteremia in those without this level of CoNS in the stool (*P* = .028). Similar findings were noted with a threshold of any detection of CoNS in stool (Supplementary Figure 5). We then asked if there was a difference in the *relative abundance* of CoNS in stool in those with CoNS bacteremia compared to those without bacteremia. Patients with CoNS bacteremia had more than a 4-fold higher abundance of CoNS in their stool compared to patients without bacteremia. Similar patterns were found for other gut bacteria linked to bacteremia risk (Supplementary Figure 6).

We further investigated each of the 53 episodes of CoNS bacteremia by collecting data on microbiological and clinical features. Vancomycin was used to treat bacteremia for at least 7 days in 44/53 (83%) episodes, indicating that clinicians considered these bacteremia events as significant. All bottles in a set were positive in 60% of collections, and more than 1 set of cultures was positive in 72% of collections. A single blood culture bottle was positive in 8/53 episodes (15%). Patients with high probability designation were more likely to have early bacteremia around the time of MBI (*P* = .017) (Supplementary Figure 7) and were more likely to have CoNS detected in stool compared to those patients with low probability designation (*P* = .002) (Supplementary Figure 8). For the 5 patients in the high probability group who experienced late CoNS bacteremia after 30 days, all demonstrated evidence of MBI at the time of bacteremia and had CoNS in their stool.

## DISCUSSION

One of the most significant adverse outcomes of HCT is bacteremia, associated with a mortality rate of 10–30% [12]. Efforts to reduce bacteremia have included prophylactic/empiric antibiotics, chlorhexidine bathing [13], and use of comprehensive central line bundles [14]. However, a key opportunity in prevention of BSIs is predicting which patients will develop bacteremia, allowing for targeted prophylactic antimicrobial therapy. Previous studies show that the gut microbiota may hold promise for predicting health outcomes post-HCT, including bacteremia and overall survival, but it is unclear how knowledge of the gut microbiota can be optimized for clinical impact in this setting.

One aim of this study was to investigate the temporal association between HCT, gut microbiota composition, and bacteremia. Bacteremia was most common in the first 2 weeks after HCT with a peak around day 9, concordant with other studies [15–17]. This window of elevated risk represents the period of neutropenia and maximal cytotoxic damage to the intestinal mucosa from conditioning chemo-radiotherapy. In our secondary analyses among patients with GvHD and patients with CoNS bacteremia during the neutropenic timeframe, the PPV increases for some organisms, suggesting that MBI may be a driver of bacteremia events, and the gut is a possible source. Cord blood recipients, who typically experience a longer period of neutropenia [18], had a much higher rate of bacteremia compared to BMT or PBSC recipients, emphasizing that this window of risk is heavily dependent on time to engraftment. A similar pattern of higher bacteremia rates was seen among ALL patients who often have multiple courses of intensive chemotherapy prior to HCT. This timeframe is also when patients experience the greatest decrease in gut microbial diversity. The combination of neutropenia, gut barrier disruption, and decreased gut microbial diversity may create a “perfect storm” for bacteremia. We found that patients who experience bacteremia have a larger decline in gut bacterial diversity than those without bacteremia events. We recognize that patients who develop bacteremia receive more broad-spectrum antibiotics, likely leading to overall lower bacterial gut diversity post treatment.

The optimal definition of intestinal domination for predicting bacteremia has not been firmly established, though previous research employed a 30% relative abundance cutoff [19]. One goal of our study was to determine the PPV of intestinal domination for predicting bacteremia at various levels of domination for each bacterium causing bacteremia. Clinicians could employ information based on a high PPV to change antibiotic therapy and prevent bacteremia. Our results indicate that for common BSIs seen in HCT patients, there is a relatively low PPV not only at 30% relative abundance, but with presence/absence, 10%, and 50% relative abundance. Even at the highest PPV (50% PPV for presence of *S. aureus*), this still only represents a 50:50 chance that a patient will go on to develop bacteremia with *S. aureus* if detected in their stool. Although several studies have demonstrated a significant association between intestinal domination and bacteremia risk in patients with cancer as reflected by elevated odds or risk ratios [2, 7], the more actionable metric for clinicians is the PPV.

Our results also indicate there may be a portion of BSIs due to MBI that are being misclassified according to current National Healthcare Safety Network (NHSN) guidelines [3], which exclude common commensal organisms. CoNS are not typically considered members of the gut microbiota. In fact, there were zero 16S rRNA sequence reads of CoNS in the stool of the 50 healthy donors included in our parent study. However, our data and that of others not only suggest that gut domination by CoNS is possible in patients with cancer [20], but that domination is associated with CoNS bacteremia. Some of our study participants had persistent CoNS present in stool with a relative abundance up to 100%. Unexpectedly, the highest PPV was seen for CoNS and *S. aureus*, 2 species that have not been the primary focus in other studies of bacteremia post-HCT [8, 16], suggesting the gut may be one of many sources of *Staphylococcus* BSI.

Further evidence of the gut as an infection source is demonstrated by patients with a high probability of true CoNS bacteremia events late in the transplant period. All patients demonstrated clinical evidence of MBI, such as steroid-refractory GvHD or chemotherapy for relapsed malignancy, that coincided with CoNS bacteremia events. We are not suggesting that all CoNS infections arise from the gut; rather, many CoNS BSIs are likely due to central line infection or skin contamination, with gut translocation representing a subset of events in cancer patients. The process for a true CoNS translocation may rely on a 2-hit scheme in which a patient not only has CoNS gut colonization but also substantial MBI.

Even if domination is not achieved by some organisms in the gut, their presence in the setting of a dysfunctional mucosal barrier may be enough to seed the bloodstream and cause an infection [21]. We determined that 67/114 (59%) bacteremia events were caused by a bacterial species that was found in the gut within 30 days of the bacteremia event, further highlighting the importance of the gut reservoir. Our findings suggest that by excluding common commensals from the NHSN list of organisms eligible for MBI designation, there may be a misclassification of bacteremia events in patients receiving myelosuppressive chemotherapy or HCT when gut translocation may be the source. Without considering CoNS in the GI tract, for example, some BSI may be inappropriately classified as hospital-based Central-Line Associated Bloodstream Infections (CLABSIs) that penalize institutions caring for these high-risk populations.

The presence of certain bacteria in the gut was positively associated with bacteremia risk, particularly bacterial genera that were responsible for BSIs (*Escherichia* and *Enterococcus*). Interestingly, other genera not implicated in any bacteremia events, such as *Lactobacillus*, were positively associated with bacteremia as well. Perhaps organisms such as *Lactobacillus* may serve as indicators of gut microbial disruption and increased bacteremia risk despite not being involved in BSIs. We found that some bacteria, such as *Roseburia*, *Blautia*, and *Anaerobutyricum*, were associated with lower risk of bacteremia. Whether this effect is achieved by maintaining gut barrier integrity, out-competing pathogenic species for nutrients, or other processes remains unclear. Investigating the potentially beneficial role of these bacteria may be useful in developing live biotherapeutics and tailoring antibiotic therapy to preserve these anaerobes in patients undergoing HCT. Similarly, recognizing bacteria that are positively associated with bacteremia risk emphasizes the importance of preserving gut microbiota composition with optimal antibiotic stewardship. The connection between antibiotics administered and the gut microbiota after HCT is outside the scope of this work, though future studies will investigate this relationship.

One of the limitations of our study is the resolution of our sequencing data. We targeted the V3–V4 region of the bacterial 16S rRNA gene. This amplicon is not as robust at distinguishing between species of *Staphylococcus*, *Streptococcus*, and *Lactobacillus* compared to the V1–V2 region. Conversely, it is well-suited for the classification of many taxa found in the gut. Some analyses were done at the genus level where additional taxonomic resolution would not have been helpful. Another limitation of our study is the stool collection frequency. Although patients attempted to collect stool samples weekly, this goal was not always met. Important shifts in microbiota composition may have been missed that could be integral to determining domination events and diversity at specific time points. Additionally, the low frequencies associated with some types of bacteremia events may lead to artificial inflation of PPV, though future studies with larger populations would be helpful to confirm these data. While we did not have the ability to directly compare bacterial strains found in the bloodstream to organisms in the stool via sequencing methods, this provides an avenue for future research to determine the role of direct translocation from the gut to the bloodstream, especially for CoNS bacteremia events. Opportunities to improve the ability of the gut microbiota to predict bacteremia in the setting of HCT include modeling additional factors such as neutropenia and evidence of gut barrier injury via biomarkers.

In conclusion, our results confirm that the gut microbiota is predictive for bacteremia events in allogeneic HCT recipients, but the PPV for most bacteria, including *Enterococcus*, *Klebsiella*, and *E. coli*, is low. The low PPV for intestinal domination as a predictor of bacteremia limits the ability to translate this knowledge into actions such as personalized antibiotic modifications. Intestinal domination by CoNS and its link with CoNS bacteremia in HCT recipients highlights a possible newly recognized source of bacterial translocation and bacteremia risk. While frequent stool sampling may not provide actionable data in terms of predicting bacteremia risk, developing live biotherapeutic products with protective species may serve as an alternative option for reducing bacteremia.

## Supplementary Material

jiag005_Supplementary_Data

## Supplementary Data

Supplementary materials are available at *The Journal of Infectious Diseases* online (http://jid.oxfordjournals.org/). Supplementary materials consist of data provided by the author that are published to benefit the reader. The posted materials are not copyedited. The contents of all supplementary data are the sole responsibility of the authors. Questions or messages regarding errors should be addressed to the author.
