# Supplementary material for: Gut Microbiota and Intestinal Monodomination as a Predictor for Bacteremia in Allogeneic Hematopoietic Cell Transplant Recipients
Source: J Infect Dis. 2026 Feb 24;234(1):e81–9. doi: 10.1093/infdis/jiag005 (PMC13431778; doi:10.1093/infdis/jiag005)
Supplement: jiag005_Supplementary_Data [file jiag005_supplementary_data.zip › Gut_Microbiota_and_Bacteremia_Supplementary.docx]

**SUPPLEMENTARY MATERIALS**

**Supplementary Methods**

DNA extraction

Stool swabs were processed with the QIAamp BiOstic Bacteremia DNA Kit (Qiagen, Germantown, Maryland). DNA was eluted in 150ul of 0.1X Tris-EDTA buffer and stored at -80°C. Negative control swabs were included in each DNA extraction to monitor for contamination in extraction reagents.

Quantification of intestinal bacteria by qPCR

All samples underwent internal amplification control (IAC) qPCR to verify the absence of PCR inhibitors [22]. The concentration of bacteria within each sample was calculated using broad range qPCR targeting the V3-V4 region of the 16S rRNA gene as previously described [23]. No-template controls were included to ensure lack of contaminants in the qPCR reagents.

Broad-range PCR, Sequencing and Processing of Sequence Reads from Stool Samples

16S rRNA gene PCR was performed on stool DNA samples using primers 338F-806R, followed by a bead clean step with Agencourt AMPUre XP Beads (Beckman Coulter, Indianapolis, Indiana) to remove primer dimers, as previously described [23]. Index PCR was performed on the bead-cleaned samples with NexteraXT index kits, followed by another bead clean step. The final bead-cleaned PCR products were eluted in 50ul 1X Tris-EDTA buffer. Individual samples were quantified using the Qubit instrument, diluted to equimolar quantities, and pooled to a total dilution of 8nM. The Illumina MiSeq instrument (Illumina, San Diego, California) was utilized to sequence stool DNA samples as previously described [23]. No-template controls and DNA extraction controls were included in the broad-range 16S rRNA gene PCRs and sequenced to monitor for contaminants. A mock community purchased from ATCC (https://www.atcc.org/products/msa-1003) with known bacterial composition was used as a positive control to evaluate if bacterial taxa present in this community were detected with our laboratory process and bioinformatics pipeline. We confirmed that all bacterial taxa in the mock community above 0.02% were detected except *Cutibacterium acnes*. There is one mismatch in the reverse primer used in our study at the 3' end of the V3-V4 region of the 16S rRNA gene sequence of *C. acnes* ATCC 11828 in the mock community which may impact amplification efficiency (Supplementary Table 9, Tab A). Raw sequence reads were demultiplexed and processed as previously described [11]. A list of unique sequence variants (SVs) was generated using the DADA2 package which was used for error correction, dereplication, paired-end assembly, and chimera removal [24]. Sequence reads are available from the NCBI Short Read Archive (Accession number: Bioproject PRJNA1281039).

We applied a minimum number of reads per sample cut-off of 5000 reads. In addition, we applied a minimum read cut-off of 25 reads at the taxon level to reduce potential for contamination. In terms of group level comparisons between samples from participants with and without bacteremia, the average read counts were 29379 and 31276 for non-bacteremia and bacteremia groups, respectively. The median read counts were 27055 and 28300 for non-bacteremia and bacteremia groups, respectively.

Reference Set Creation and Taxonomic Assignment

A reference set for taxonomic assignment of sequence reads from stool samples was created as previously described [11]. Briefly, the SVs generated in the study were used to recruit full-length 16S rRNA gene sequences from NCBI and a phylogenetic tree was constructed. The list of bacterial taxa used for the creation of the gut reference set used for our analyses is provided in Supplementary Table 9. Taxonomy of each unique SV was assigned based on location on the phylogenetic tree. Taxonomic assignments were validated by inspecting multiple sequence alignments, phylogenetic trees and BLAST searches. Bacterial taxa represented by fewer than 25 reads were excluded from a sample to minimize environmental contaminant sequences from being included in the final dataset. Sequences judged to be contaminants were filtered from the final dataset (Supplementary Table 9, Tab D).

Statistical Methods

All statistical analyses were performed using R version 4.3.1 [25]. Statistical significance was set at p ≤ 0.05. To evaluate the association between bacterial genera and bacteremia, we performed t-test comparisons of genus-level relative abundance for bacteremia patients v. non-bacteremia patients. We averaged the genus-level relative abundance among all samples for a given patient before comparing one averaged value per taxa for each group. To formally compare groups over time for Figure 2, we divided the study timeline into rolling windows of fixed duration (e.g., 10 days wide, stepped every 3 days). Within each window, we performed a nonparametric Wilcoxon rank-sum test to compare Shannon diversity between samples from participants with Bacteremia vs participants without Bacteremia. Only windows that contained observations from both groups were tested. The resulting window-specific p-values were mapped back onto the time axis and visualized as yellow shaded intervals, highlighting specific temporal regions where groups differed significantly. Overall survival (Figure 3) was estimated using a Kaplan-Meier survival curve. The p-value is from a Cox Proportional-Hazards model (via coxph function in R) adjusted for age and sex.

Determining Probability of True CoNS Bacteremia Events and Defining Mucosal Barrier Injury

Patients were deemed to have a high probability of true CoNS bacteremia if they had at least 2 bottles positive in a set, and at least 2 sets positive for this organism in a 3-day period. Patients deemed to have low probability of true CoNS bacteremia had only 1 bottle of a blood culture set positive, or only one set positive if multiple bottles were positive in a set. Blood culture sets consisted of anaerobic, aerobic, and sometimes fungal culture bottles. When we evaluated the four patients (with five events) who had a high probability of true CoNS bacteremia between day 35 and 112 after transplant (late onset), mucosal barrier injury was defined as either presence of active GI GvHD on steroids, within 2 weeks of re-induction chemotherapy, or with a diagnosis of colitis and mucositis within 3 weeks prior to bacteremia.

**Supplementary References**

22. Khot PD, Ko DL, Hackman RC, Fredricks, DN. Development and optimization of quantitative PCR for the diagnosis of invasive aspergillosis with bronchoalveolar lavage fluid. *BMC Infect Dis*, **2008**; 8: 73.

23. Golob J, Pergam S, Srinivasan S, et al. Stool Microbiota at Neutrophil Recovery Is Predictive for Severe Acute Graft vs Host Disease After Hematopoietic Cell Transplantation. *Clin Infect Dis*, **2017**; 65: 1984-1991.

24. Callahan BJ, McMurdie PJ, Rosen MJ, Han AW, Johnson AJ, Holmes SP. DADA2: High-resolution sample inference from Illumina amplicon data. *Nat Methods*, **2016**; 13: 581-583.

25. R Core Team. R: A language and environment for statistical computing. Vienna, Austria: R Foundation for Statistical Computing, 2023; Version 4.3.1. [https://www.R-project.org](https://www.r-project.org/)
