# Supplementary material for: Gut Microbiota and Intestinal Monodomination as a Predictor for Bacteremia in Allogeneic Hematopoietic Cell Transplant Recipients
Source: J Infect Dis. 2026 Feb 24;234(1):e81–9. doi: 10.1093/infdis/jiag005 (PMC13431778; doi:10.1093/infdis/jiag005)

Supplementary Figure 2. Frequency of Bacterial Organisms in Bacteremia Events. A complete list of the 130 bacteremia events organized by bacterial species and number of episodes.

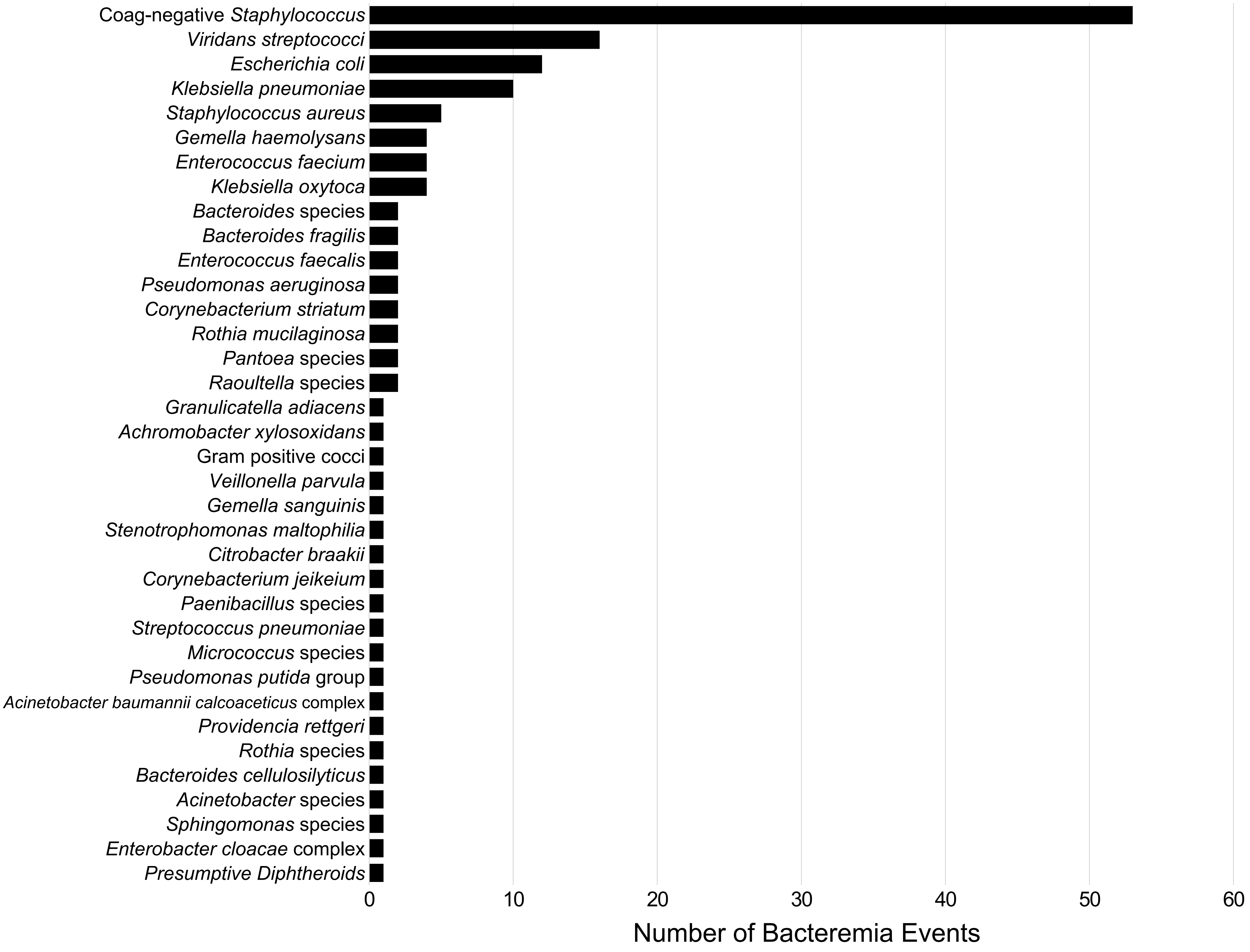

Supplement: jiag005_Supplementary_Data [file jiag005_supplementary_data.zip › Supplementary_Figure_02.pdf]
