# Supplementary material for: Gut Microbiota and Intestinal Monodomination as a Predictor for Bacteremia in Allogeneic Hematopoietic Cell Transplant Recipients
Source: J Infect Dis. 2026 Feb 24;234(1):e81–9. doi: 10.1093/infdis/jiag005 (PMC13431778; doi:10.1093/infdis/jiag005)

Supplementary Figure 3. Gut Microbial Diversity Without Post-Bacteremia Samples and Divided by Timing of Bacteremia Onset. Utilizing the same methods as Figure 2, we repeated our analysis and excluded any samples collected after a bacteremia event (A) and compared gut diversity among patients who had bacteremia within 14 days of transplant (colored in orange) or bacteremia more than 14 days post-transplant (colored in purple) (B).

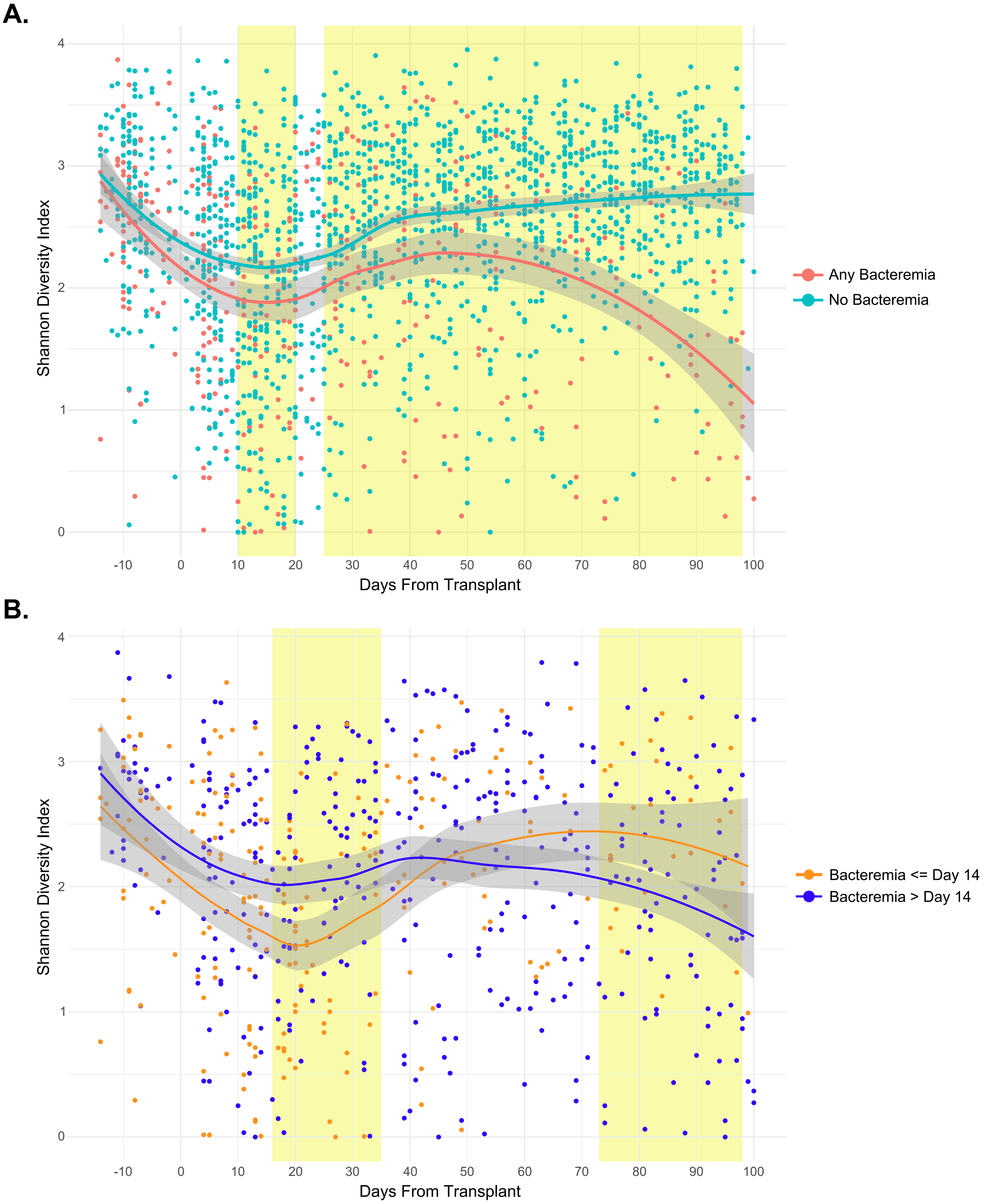

Supplement: jiag005_Supplementary_Data [file jiag005_supplementary_data.zip › Supplementary_Figure_03.pdf]
