# Supplementary material for: Gut Microbiota and Intestinal Monodomination as a Predictor for Bacteremia in Allogeneic Hematopoietic Cell Transplant Recipients
Source: J Infect Dis. 2026 Feb 24;234(1):e81–9. doi: 10.1093/infdis/jiag005 (PMC13431778; doi:10.1093/infdis/jiag005)

Supplementary Figure 4. Genus level identification of stool microbiota. A total of 694,391,140 16S rRNA gene sequence reads were obtained from 2,316 stool swabs, with a mean of 29,982 reads per stool sample, minimum of 5,124 reads and maximum of 158,941 reads.

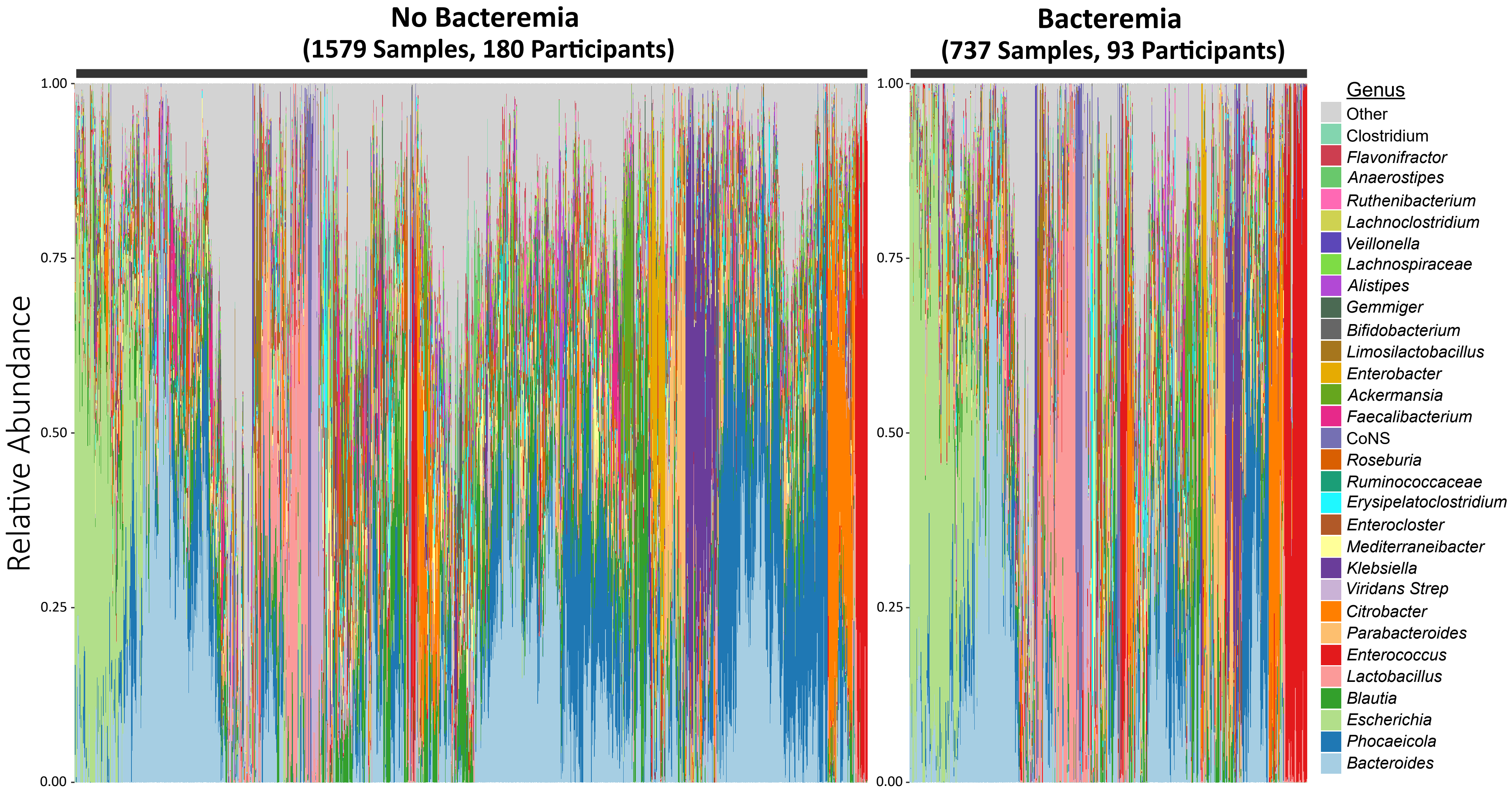

Supplement: jiag005_Supplementary_Data [file jiag005_supplementary_data.zip › Supplementary_Figure_04.pdf]
