# Supplementary material for: Gut Microbiota and Intestinal Monodomination as a Predictor for Bacteremia in Allogeneic Hematopoietic Cell Transplant Recipients
Source: J Infect Dis. 2026 Feb 24;234(1):e81–9. doi: 10.1093/infdis/jiag005 (PMC13431778; doi:10.1093/infdis/jiag005)

Supplementary Figure 5. Relative Abundance of Coagulase-negative *Staphylococcus* Species in the Stool. Patients who experienced at least one episode of CoNS bacteremia had a higher average number of samples with CoNS in the gut compared to other patients who had non-CoNS bacteremia events and patients who never experienced a bacteremia event. This pattern was true when examining samples that contained at least 30% relative abundance of CoNS (A) and > 0% CoNS (B).

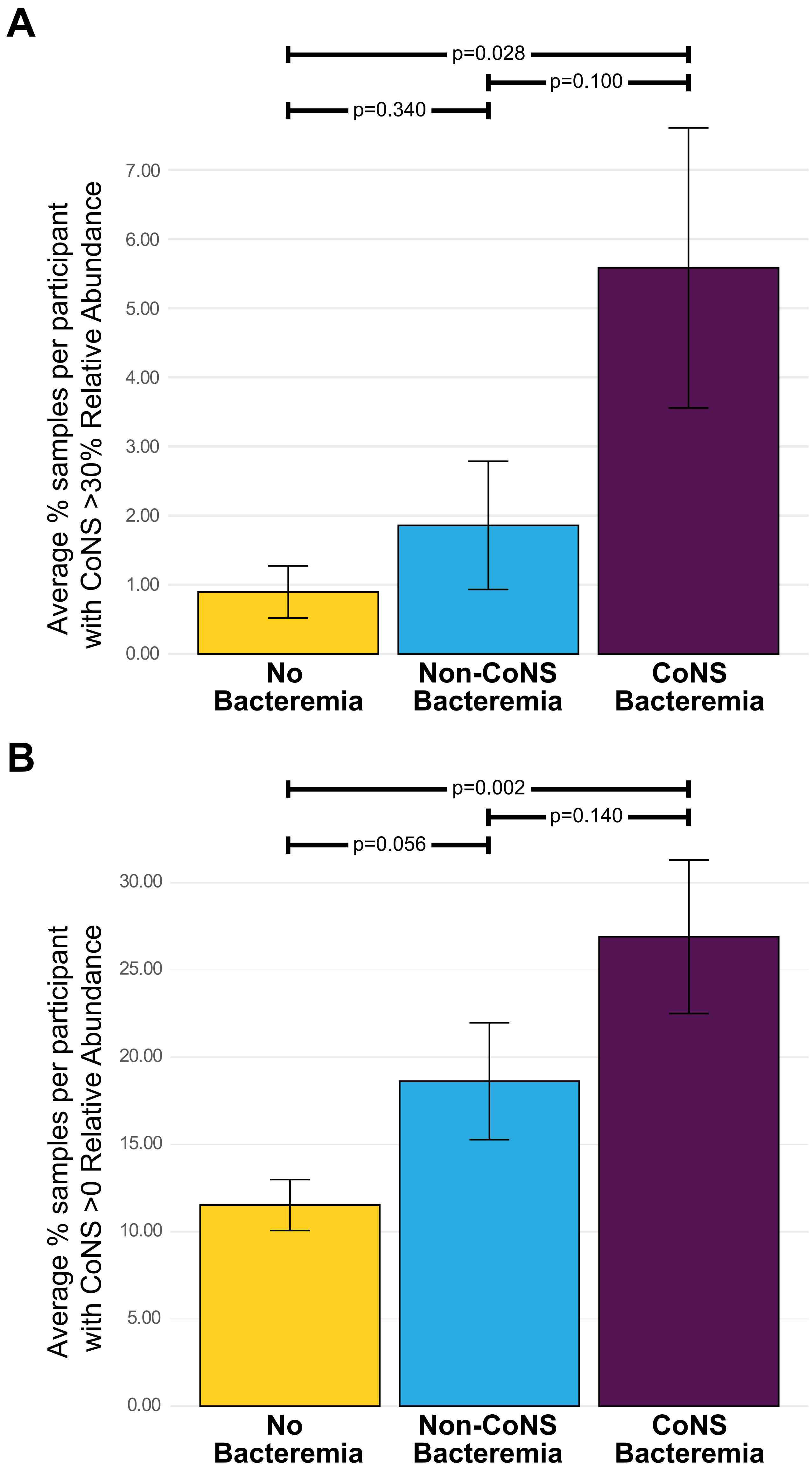

Supplement: jiag005_Supplementary_Data [file jiag005_supplementary_data.zip › Supplementary_Figure_05.pdf]
