# Supplementary material for: Gut Microbiota and Intestinal Monodomination as a Predictor for Bacteremia in Allogeneic Hematopoietic Cell Transplant Recipients
Source: J Infect Dis. 2026 Feb 24;234(1):e81–9. doi: 10.1093/infdis/jiag005 (PMC13431778; doi:10.1093/infdis/jiag005)

Supplementary Figure 6. Average Relative Abundance of Bacteremia Species Found in Stool. For some of the most common bacterial species implicated in bacteremia events, there was an increased relative abundance of these same bacterial species found in stool samples of patients who experienced bacteremia compared to patients without bacteremia. *Gemella hemolysans* and *Staphylococcus aureus* were excluded due to low overall relative abundance in all samples. (A) CoNS (p = 0.045), (B) *E. coli* (p = 0.069), (C) *Enterococcus* (p = 0.085), (D) *Klebsiella* (p = 0.280), (E) *Viridans streptococci* (p = 0.360)

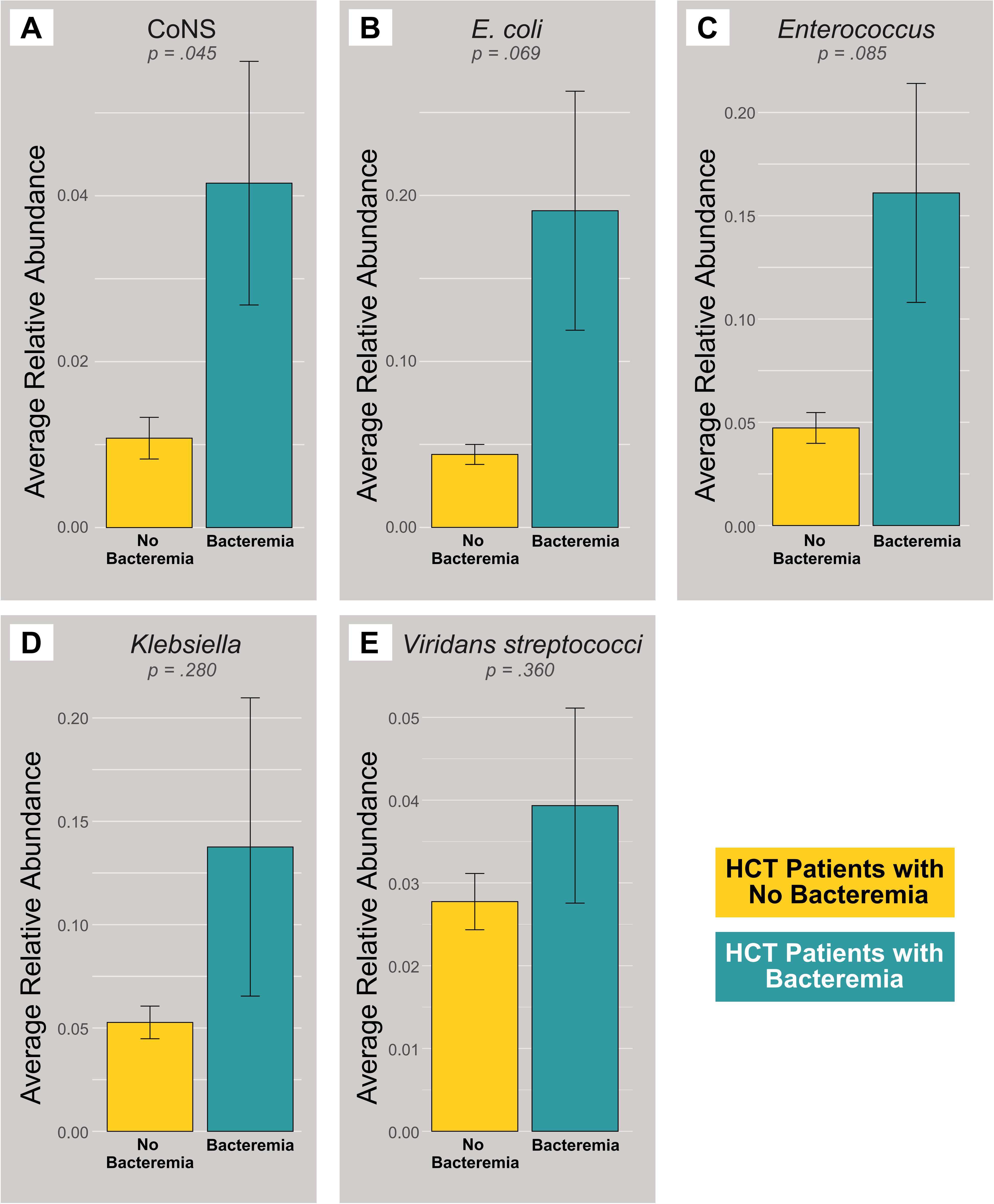

Supplement: jiag005_Supplementary_Data [file jiag005_supplementary_data.zip › Supplementary_Figure_06.pdf]
