# Supplementary material for: Gut Microbiota and Intestinal Monodomination as a Predictor for Bacteremia in Allogeneic Hematopoietic Cell Transplant Recipients
Source: J Infect Dis. 2026 Feb 24;234(1):e81–9. doi: 10.1093/infdis/jiag005 (PMC13431778; doi:10.1093/infdis/jiag005)

Supplementary Figure 7. Temporal Association between CoNS Bacteremia Event and Probability of True CoNS Bacteremia Event. Of the 53 bacteremia events involving CoNS species, 20 events were deemed high probability to be a true CoNS bacteremia event based on multiple positive blood culture sets. These events were more likely to occur (p = .017) early in the post-transplant period compared to low probability events (when only a single blood culture set was positive).

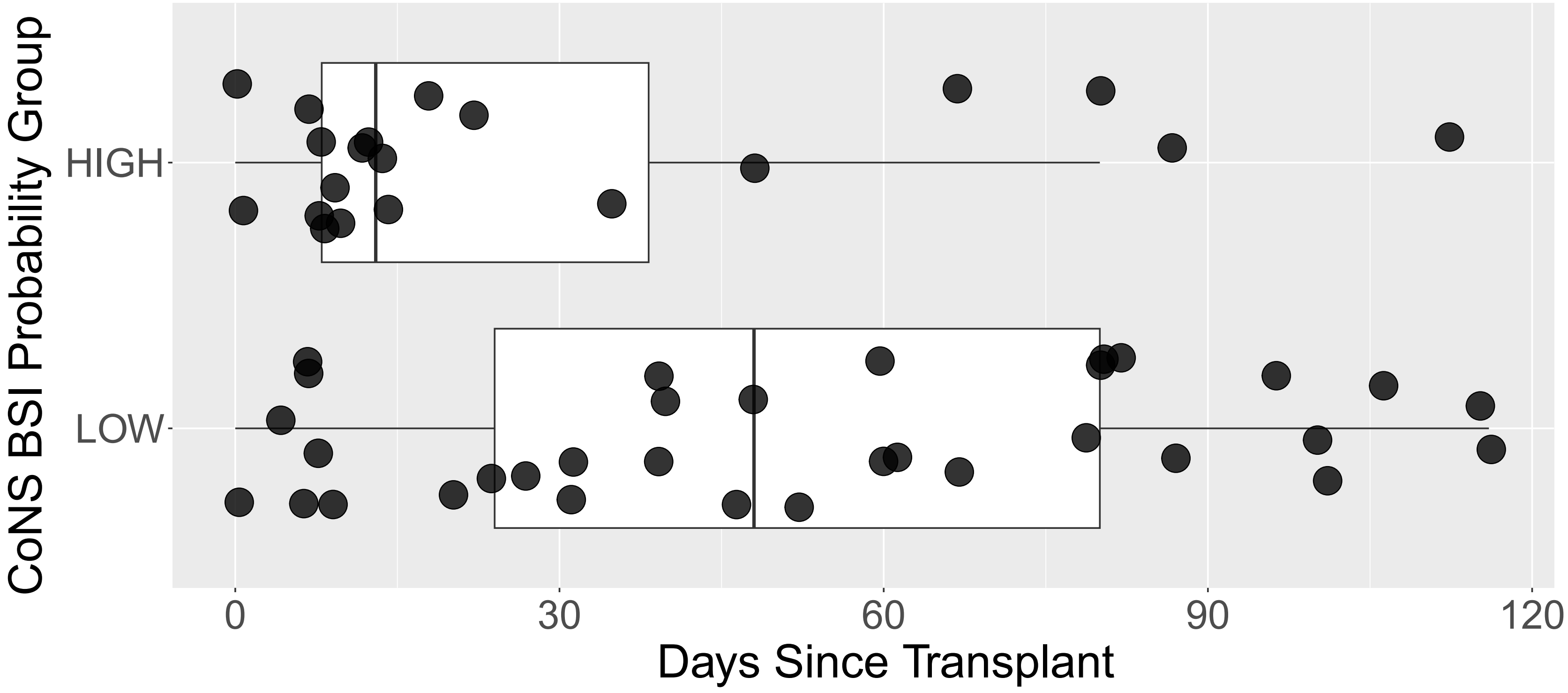

Supplement: jiag005_Supplementary_Data [file jiag005_supplementary_data.zip › Supplementary_Figure_07.pdf]
