# Supplementary material for: Gut Microbiota and Intestinal Monodomination as a Predictor for Bacteremia in Allogeneic Hematopoietic Cell Transplant Recipients
Source: J Infect Dis. 2026 Feb 24;234(1):e81–9. doi: 10.1093/infdis/jiag005 (PMC13431778; doi:10.1093/infdis/jiag005)

Supplementary Figure 8. Presence of CoNS in the Gut and Probability of True CoNS Bacteremia Event. We examined the stool samples from each patient who had a CoNS bacteremia event to determine if there were ever any CoNS sequencing reads. Using the same system as described in Supplementary Figure 7 to determine high v. low probability of a true CoNS bacteremia event (multiple v. single positive blood culture set), we found that patients with a high probability of a true CoNS bacteremia event were significantly more likely to have CoNS found in their stool than patients with a low probability of a true CoNS bacteremia event ($p = 0.002$). All 4 patients (5 bacteremia events) with a high probability of true CoNS bacteremia who experienced bacteremia after 30 days post-transplant also had evidence of mucosal barrier injury at the time of bacteremia.

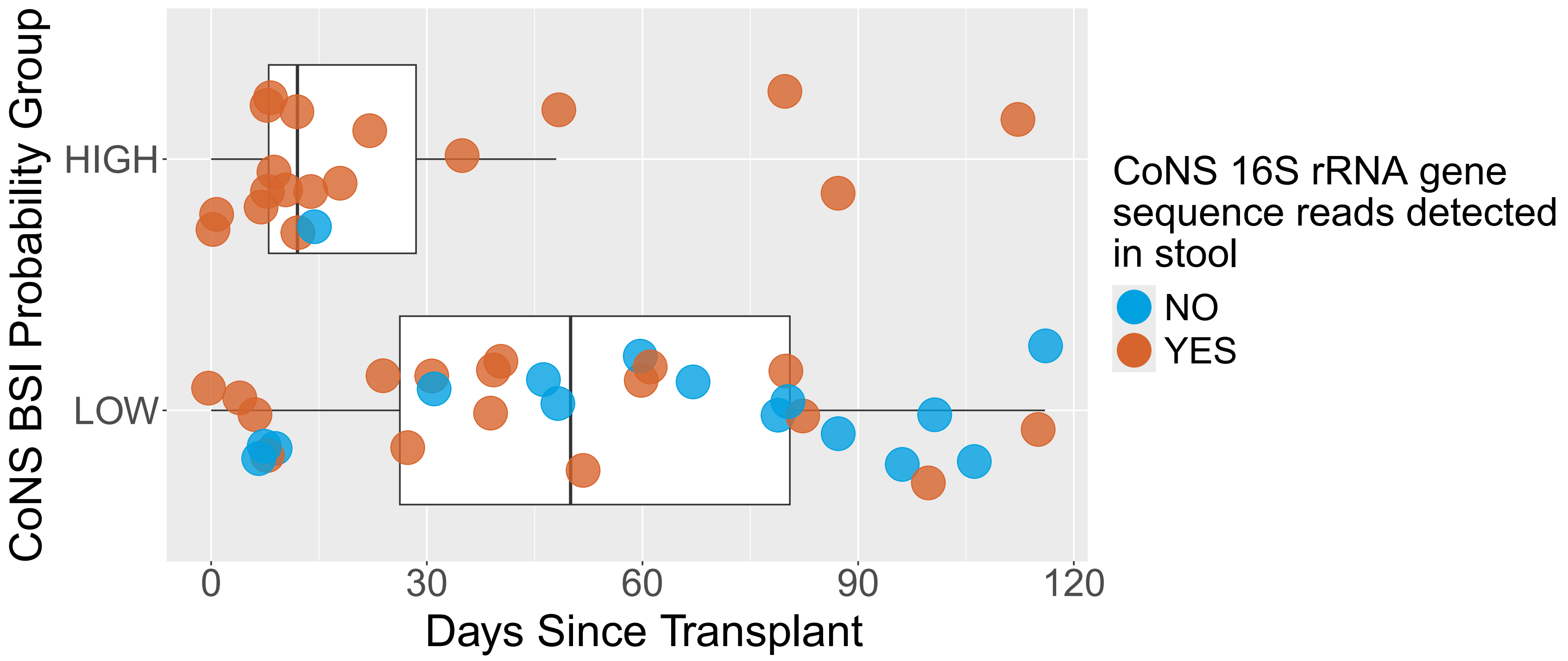

Supplement: jiag005_Supplementary_Data [file jiag005_supplementary_data.zip › Supplementary_Figure_08.pdf]
