# Supplementary material for: Gut Microbiota and Intestinal Monodomination as a Predictor for Bacteremia in Allogeneic Hematopoietic Cell Transplant Recipients
Source: J Infect Dis. 2026 Feb 24;234(1):e81–9. doi: 10.1093/infdis/jiag005 (PMC13431778; doi:10.1093/infdis/jiag005)
Supplement: jiag005_Supplementary_Data [file jiag005_supplementary_data.zip › Supplementary_Table_02.pdf]

Supplementary Table 2. Characterization of Bacteremia Events

		Events (n = 130)
**Gram stain**		
	Gram positive	81 (62.3%)
	Gram negative	35 (26.9%)
	Mixed	14 (10.8%)
**Oxygen requirements**		
	Aerobe	10 (7.7%)
	Anaerobe	4 (3.1%)
	Facultative anaerobe	101 (77.7%)
	Mixed	14 (10.8%)
	Other	1 (0.8%)
**Monomicrobial v. polymicrobial bacteremia events**		
	Monomicrobial events	116 (89.2%)
	Polymicrobial events	14 (10.8%)
		**Subjects (n = 95)**
**Number of bacteremia events per patient**		
	1	70 (73.7%)
	2	16 (16.8%)
	≥3	9 (9.5%)
