# Supplementary material for: Gut Microbiota and Intestinal Monodomination as a Predictor for Bacteremia in Allogeneic Hematopoietic Cell Transplant Recipients
Source: J Infect Dis. 2026 Feb 24;234(1):e81–9. doi: 10.1093/infdis/jiag005 (PMC13431778; doi:10.1093/infdis/jiag005)
Supplement: jiag005_Supplementary_Data [file jiag005_supplementary_data.zip › Supplementary_Table_03.pdf]

Supplementary Table 3.Most Common Gut Bacterial Species Among CBT Recipients and ALL Patients. Top bacterial genera comparing A) Stool samples from cord blood transplant (CBT) recipients and non-cord blood transplant recipients and B) Stool samples from patients with underlying Acute Lymphoblastic Leukemia (ALL), Acute Myeloid Leukemia (AML), and patients with other underlying conditions. Only genera exceeding 1% relative abundance threshold in at least one group are shown.

A

Genus	Samples From CBT Transplant Recipients	Samples From Non-CBT Transplant Recipients
Escherichia	17.7%	5.9%
Bacteroides	10.4%	13.0%
Enterococcus	7.7%	4.1%
Staphylococcus CoNS	6.9%	1.0%
Lactobacillus	6.2%	5.2%
Phocaeicola	4.8%	10.6%
Klebsiella	4.8%	2.7%
Akkermansia	2.8%	1.1%
Veillonella	2.5%	0.8%
Blautia	2.5%	6.5%
Citrobacter	2.3%	3.6%
Parabacteroides	2.2%	4.4%
Viridans streptococci	1.9%	3.0%
Erysipelatoclostridium	1.8%	1.9%
Enterocloster	1.6%	2.1%
Mediterraneibacter	1.3%	2.2%
Lachnospiraceae	1.0%	0.9%
Bifidobacterium	0.9%	1.0%
Gemmiger	0.9%	1.0%
Limosilactobacillus	0.9%	1.0%
Alistipes	0.8%	1.0%
Ruminococcaceae	0.8%	1.6%
Faecalibacterium	0.8%	1.4%
Roseburia	0.5%	1.6%
Enterobacter	0.3%	1.1%

B

Genus	Samples From Participants with ALL	Samples From Participants with AML	Samples From Participants with No ALL or AML
Bacteroides	13.0%	10.2%	14.2%
Phocaeicola	6.4%	9.5%	11.0%
Escherichia	5.7%	7.3%	6.7%
Blautia	5.3%	6.7%	6.1%
Lactobacillus	5.8%	6.3%	4.6%
Parabacteroides	4.9%	3.7%	4.3%
Klebsiella	6.2%	3.3%	2.2%
Citrobacter	4.3%	4.1%	3.0%
Enterococcus	1.3%	4.8%	4.6%
Viridans streptococci	5.2%	2.5%	2.8%
Erysipelatoclostridium	2.7%	2.4%	1.5%
Mediterraneibacter	2.2%	1.8%	2.2%
Enterocloster	1.6%	2.3%	2.0%
Roseburia	1.6%	1.7%	1.5%
Ruminococcaceae	1.2%	1.7%	1.5%
Faecalibacterium	1.7%	0.9%	1.5%
Staphylococcus CoNS	0.8%	1.9%	1.3%
Bifidobacterium	1.7%	0.8%	1.0%
Enterobacter	1.1%	1.3%	0.9%
Akkermansia	0.7%	1.3%	1.3%
Veillonella	1.2%	1.1%	0.8%
Ruthenibacterium	1.3%	1.0%	0.8%
Lachnospiraceae	1.0%	0.9%	1.0%
Clostridium	1.2%	0.9%	0.7%
Gemmiger	0.8%	0.8%	1.1%
Anaerostipes	1.2%	0.6%	0.9%
Limosilactobacillus	0.6%	1.0%	1.1%
Pediococcus	1.3%	1.1%	0.3%
Alistipes	0.6%	0.9%	1.1%
Flavonifractor	0.6%	1.0%	0.7%
Terrisporobacter	1.1%	0.4%	0.3%
