# Supplementary material for: Gut Microbiota and Intestinal Monodomination as a Predictor for Bacteremia in Allogeneic Hematopoietic Cell Transplant Recipients
Source: J Infect Dis. 2026 Feb 24;234(1):e81–9. doi: 10.1093/infdis/jiag005 (PMC13431778; doi:10.1093/infdis/jiag005)
Supplement: jiag005_Supplementary_Data [file jiag005_supplementary_data.zip › Supplementary_Table_04.pdf]

Supplementary Table 4. Bacterial Genera Associated with Bacteremia Risk. Participant level mean relative abundance was compared between patients with bacteremia and those with no bacteremia using a two-sided t-test. The top three genera associated with bacteremia are highlighted in blue (positive association) and green (negative association).

Genus	**Mean Relative Abundance Bacteremia Group**	**Mean Relative Abundance No Bacteremia Group**	**p-value**	**Adjusted p-value**	**Association Direction**
Roseburia	0.0070	0.0193	1.7801E-07	4.06E-05	NEGATIVE
Blautia	0.0424	0.0683	1.1155E-05	0.0013	NEGATIVE
Anaerobutyricum	0.0027	0.0057	0.0001	0.0071	NEGATIVE
Intestinibacter	0.0010	0.0033	0.0006	0.0314	NEGATIVE
Lachnospiraceae/Eubacteriaceae	4.3E-07	4.8E-05	0.0013	0.0586	NEGATIVE
Peptococcaceae	9.2E-07	0.0001	0.0050	0.1131	NEGATIVE
Erysipelotrichaceae	3.3E-06	4.7E-05	0.0043	0.1131	NEGATIVE
Negativibacillus	0.0010	0.0025	0.0036	0.1131	NEGATIVE
Hungatella	0.0013	0.0032	0.0031	0.1131	NEGATIVE
Sellimonas	0.0015	0.0028	0.0045	0.1131	NEGATIVE
Gordonibacter	8.2E-06	0.0001	0.0163	0.2028	NEGATIVE
Adlercreutzia/Asaccharobacter	0.0001	0.0003	0.0106	0.2028	NEGATIVE
Intestinimonas	0.0002	0.0005	0.0160	0.2028	NEGATIVE
Evtepia	0.0003	0.0005	0.0139	0.2028	NEGATIVE
Lachnospira	0.0013	0.0025	0.0121	0.2028	NEGATIVE
Dorea	0.0042	0.0074	0.0178	0.2028	NEGATIVE
Lachnoclostridium	0.0069	0.0094	0.0137	0.2028	NEGATIVE
Faecalibacterium	0.0086	0.0157	0.0166	0.2028	NEGATIVE
Staphylococcus CoNS	0.0285	0.0088	0.0177	0.2028	POSITIVE
Enterococcus	0.0794	0.0344	0.0126	0.2028	POSITIVE
Coprococcus	0.0015	0.0026	0.0198	0.2050	NEGATIVE
Lachnospiraceae/Clostridiaceae	0.0019	0.0037	0.0191	0.2050	NEGATIVE
Odoribacter	0.0001	0.0005	0.0226	0.2243	NEGATIVE
Turicibacter	0.0001	0.0001	0.0244	0.2314	NEGATIVE
Lachnospiraceae	0.0073	0.0107	0.0265	0.2412	NEGATIVE
Lactobacillus	0.0670	0.0424	0.0283	0.2482	POSITIVE
Escherichia	0.0736	0.0376	0.0301	0.2538	POSITIVE
Oscillibacter	0.0002	0.0004	0.0326	0.2653	NEGATIVE
Colidextribacter	1.3E-05	0.0001	0.0359	0.2731	NEGATIVE
Holdemania	1.7E-05	4.6E-05	0.0358	0.2731	NEGATIVE
Parasutterella	0.0006	0.0023	0.0381	0.2803	NEGATIVE
Anaeromassilibacillus	0.0007	0.0013	0.0394	0.2809	NEGATIVE
Eggerthellaceae	0.0001	0.0003	0.0436	0.3010	NEGATIVE
Caprobacter	0	4.7E-06	0.0462	0.3046	NEGATIVE
Eisenbergiella	0.0011	0.0027	0.0481	0.3046	NEGATIVE
Tyzzerella/Coprococcus	0.0019	0.0034	0.0473	0.3046	NEGATIVE
Fusicatenibacter	0.0014	0.0029	0.0516	0.3132	NEGATIVE
Dysosmobacter	0.0030	0.0040	0.0522	0.3132	NEGATIVE
Massilimaliae	3.0E-05	0.0001	0.0559	0.3150	NEGATIVE
Butyricimonas	0.0002	0.0007	0.0579	0.3150	NEGATIVE
Romboutsia	0.0006	0.0009	0.0589	0.3150	NEGATIVE
Clostridiales	0.0014	0.0023	0.0594	0.3150	NEGATIVE
Enterocloster	0.0164	0.0219	0.0542	0.3150	NEGATIVE
Bariatricus	3.4E-06	2.5E-05	0.0667	0.3377	NEGATIVE
Anaerostipes	0.0067	0.0100	0.0654	0.3377	NEGATIVE
Neobittarella	5.0E-05	0.0001	0.0694	0.3395	NEGATIVE
Succiniclasticum	0.0005	1.3E-05	0.0700	0.3395	POSITIVE
Ruminococcaceae	0.0132	0.0175	0.0822	0.3824	NEGATIVE
Enterobacter/Kluyvera	3.9E-05	0.0004	0.0957	0.4362	NEGATIVE
Morganella	1.1E-06	2.9E-05	0.1219	0.4712	NEGATIVE
Stomatobaculum	5.2E-06	0.0001	0.1186	0.4712	NEGATIVE
Christensenella	2.5E-05	0.0001	0.1196	0.4712	NEGATIVE
Adlercreutzia	0.0001	0.0002	0.1116	0.4712	NEGATIVE

Genus	Mean Relative Abundance Bacteremia Group	Mean Relative Abundance No Bacteremia Group	p-value	Adjusted p-value	Association Direction
Selenomonadales	0.0002	0.0005	0.1200	0.4712	NEGATIVE
Flintibacter	0.0012	0.0019	0.1118	0.4712	NEGATIVE
Holdemanella	0.0016	0.0005	0.1175	0.4712	POSITIVE
Gemmiger	0.0079	0.0119	0.1194	0.4712	NEGATIVE
Mediterraneibacter	0.0184	0.0239	0.1150	0.4712	NEGATIVE
Shuttleworthia	0.0001	6.2E-07	0.1283	0.4875	POSITIVE
Lancefieldella	0.0009	0.0001	0.1352	0.5052	POSITIVE
Lactimicrobium	0	0.0002	0.1459	0.5153	NEGATIVE
Anaerofustis	1.1E-05	0.0001	0.1469	0.5153	NEGATIVE
Casaltella	2.9E-05	0	0.1417	0.5153	POSITIVE
Butyricicoccus	0.0006	0.0010	0.1435	0.5153	NEGATIVE
Enterobacteriaceae	2.1E-05	0.0008	0.1536	0.5246	NEGATIVE
Olsenella	0.0004	1.0E-05	0.1542	0.5246	POSITIVE
Fournierella	0.0004	0.0011	0.1648	0.5526	NEGATIVE
Saccharofermentans	5.0E-05	0	0.1693	0.5554	POSITIVE
Monoglobus	0.0004	0.0007	0.1705	0.5554	NEGATIVE
Tyzzerella	0.0001	0.0002	0.1800	0.5782	NEGATIVE
Phocaeicola	0.0963	0.1148	0.1837	0.5818	NEGATIVE
Fenollaria	2.4E-06	0.0001	0.2026	0.5852	NEGATIVE
Hafnia/Obesumbacterium	1.2E-05	0.0001	0.1879	0.5852	NEGATIVE
Peribacillus	3.6E-05	0.0005	0.1910	0.5852	NEGATIVE
Raoultibacter	0.0001	0.0002	0.2003	0.5852	NEGATIVE
Marseillibacter	0.0001	0.0002	0.2052	0.5852	NEGATIVE
Peptostreptococcus	0.0005	5.7E-06	0.1951	0.5852	POSITIVE
Limosilactobacillus	0.0122	0.0074	0.2009	0.5852	POSITIVE
Muribaculaceae	7.0E-06	0.0003	0.2111	0.5941	NEGATIVE
Schaalia	0.0024	0.0009	0.2139	0.5947	POSITIVE
Paraclostridium	1.3E-06	4.4E-05	0.2303	0.6207	NEGATIVE
Agathobaculum/Butyricicoccus	1.2E-05	2.9E-05	0.2423	0.6207	NEGATIVE
Peptostreptococcaceae	3.7E-05	0.0005	0.2408	0.6207	NEGATIVE
Weissella	0.0018	0.0035	0.2410	0.6207	NEGATIVE
Granulicatella	0.0029	0.0010	0.2355	0.6207	POSITIVE
Viridans streptococci	0.0238	0.0308	0.2260	0.6207	NEGATIVE
Bacteroides	0.1188	0.1377	0.2403	0.6207	NEGATIVE
Dickeya	0	3.6E-07	0.3187	0.6235	NEGATIVE
Beduini	0	0.0014	0.3187	0.6235	NEGATIVE
Frankia	0	0.0028	0.3187	0.6235	NEGATIVE
Dermabacter	7.4E-07	2.4E-05	0.2602	0.6235	NEGATIVE
Rubneribacter	2.8E-06	1.1E-05	0.3000	0.6235	NEGATIVE
Hungatella/Lacrimispora	6.1E-06	0	0.3199	0.6235	POSITIVE
Leuconostoc	1.5E-05	0.0012	0.3063	0.6235	NEGATIVE
Solobacterium	2.6E-05	5.3E-06	0.3139	0.6235	POSITIVE
Dakarella/Mesosutterella	0.0001	8.0E-06	0.2664	0.6235	POSITIVE
Faecalitalea	0.0001	0.0002	0.2862	0.6235	NEGATIVE
Absiella	0.0001	0.0003	0.2641	0.6235	NEGATIVE
Merdimonas	0.0001	0.0002	0.3169	0.6235	NEGATIVE
Actinomyces	0.0002	0.0001	0.2932	0.6235	POSITIVE
Metaprevotella	0.0002	0	0.3199	0.6235	POSITIVE
Corynebacterium	0.0002	0.0007	0.2759	0.6235	NEGATIVE
Mobiluncus	0.0002	1.1E-06	0.3168	0.6235	POSITIVE
Streptococcus	0.0003	0.0006	0.2890	0.6235	NEGATIVE
Faecalicatena	0.0003	0.0001	0.2758	0.6235	POSITIVE
Rarimicrobium	0.0003	7.4E-07	0.2593	0.6235	POSITIVE
Porphyromonas	0.0004	0.0001	0.3150	0.6235	POSITIVE
Christensenellaceae	0.0005	0.0007	0.3165	0.6235	NEGATIVE
Cronobacter	0.0007	0	0.3199	0.6235	POSITIVE

Genus	Mean Relative Abundance Bacteremia Group	Mean Relative Abundance No Bacteremia Group	p-value	Adjusted p-value	Association Direction
Peptoniphilus	0.0008	0.0003	0.2953	0.6235	POSITIVE
Barnesiella	0.0018	0.0030	0.2815	0.6235	NEGATIVE
Clostridioides	0.0020	0.0011	0.2673	0.6235	POSITIVE
Pantoea/Enterobacter	0.0022	0.0005	0.2982	0.6235	POSITIVE
Collinsella	0.0025	0.0035	0.2466	0.6235	NEGATIVE
Ruminococcus	0.0035	0.0043	0.3095	0.6235	NEGATIVE
Coprobacillus	0.0007	0.0004	0.3243	0.6248	POSITIVE
Agathobaculum	0.0010	0.0013	0.3261	0.6248	NEGATIVE
Firmicutes	0.0002	6.0E-06	0.3314	0.6272	POSITIVE
Massiliprevotella	0.0002	0.0001	0.3329	0.6272	POSITIVE
Arcanobacterium	2.8E-06	1.2E-05	0.3719	0.6319	NEGATIVE
Solibacillus	2.9E-06	7.8E-06	0.3570	0.6319	NEGATIVE
Catabacter	9.1E-06	4.3E-05	0.3417	0.6319	NEGATIVE
Clostridiaceae/Ruminococcaceae	3.0E-05	4.5E-05	0.3825	0.6319	NEGATIVE
Allisonella	3.4E-05	0.0001	0.3621	0.6319	NEGATIVE
Slackia	4.9E-05	2.1E-05	0.3753	0.6319	POSITIVE
Amedibacillus	0.0001	0.0001	0.3776	0.6319	NEGATIVE
Cellulosilyticum	0.0001	0.0003	0.3713	0.6319	NEGATIVE
Emergencia	0.0001	0.0001	0.3446	0.6319	POSITIVE
Eubacteriaceae/Clostridiaceae	0.0002	0.0001	0.3738	0.6319	POSITIVE
Bifidobacteriaceae	0.0002	0.0001	0.3815	0.6319	POSITIVE
Muricomes	0.0003	0.0001	0.3798	0.6319	POSITIVE
Anaerotignum	0.0014	0.0017	0.3752	0.6319	NEGATIVE
Raoultella	0.0026	0.0012	0.3779	0.6319	POSITIVE
Acidaminococcus	0.0027	0.0014	0.3735	0.6319	POSITIVE
Terrisporobacter	0.0044	0.0063	0.3503	0.6319	NEGATIVE
Flavonifractor	0.0076	0.0089	0.3537	0.6319	NEGATIVE
Beduinibacterium	8.2E-06	2.4E-06	0.3858	0.6328	POSITIVE
Turicimonas	5.6E-06	3.8E-05	0.3998	0.6365	NEGATIVE
Lawsonella	2.8E-05	0.0002	0.4152	0.6365	NEGATIVE
Extibacter	0.0003	0.0001	0.4056	0.6365	POSITIVE
Anaerotruncus	0.0008	0.0010	0.4160	0.6365	NEGATIVE
Anaerococcus	0.0015	0.0008	0.3951	0.6365	POSITIVE
Pseudomonas	0.0027	0.0014	0.3935	0.6365	POSITIVE
Pediococcus	0.0095	0.0050	0.4025	0.6365	POSITIVE
Alistipes	0.0096	0.0120	0.4007	0.6365	NEGATIVE
Citrobacter	0.0372	0.0298	0.4104	0.6365	POSITIVE
Parabacteroides	0.0519	0.0436	0.4120	0.6365	POSITIVE
Mediterraneibacter/Blautia	0.0001	4.2E-05	0.4263	0.6366	POSITIVE
Niameybacter	0.0002	0.0003	0.4272	0.6366	NEGATIVE
Caproiciproducens	0.0002	0.0001	0.4227	0.6366	POSITIVE
Campylobacter	0.0006	0.0009	0.4269	0.6366	NEGATIVE
Faecalibacillus	0.0008	0.0012	0.4305	0.6373	NEGATIVE
Serratia	2.9E-06	6.0E-07	0.4384	0.6435	POSITIVE
Frisingicoccus	0.0002	0.0002	0.4406	0.6435	NEGATIVE
Ruthenibacterium	0.0107	0.0092	0.4431	0.6435	POSITIVE
Stenotrophomonas	0.0001	1.5E-05	0.4479	0.6463	POSITIVE
Pygmaiobacter	0.0004	0.0002	0.4571	0.6527	POSITIVE
Proteus	0.0027	0.0012	0.4581	0.6527	POSITIVE
Paraprevotella	0.0024	0.0034	0.4632	0.6560	NEGATIVE
Massilicoli	2.5E-05	4.9E-05	0.4894	0.6642	NEGATIVE
Dielma	4.3E-05	1.7E-05	0.4821	0.6642	POSITIVE
Senegalimassilia	0.0002	0.0002	0.4889	0.6642	POSITIVE
Longibaculum	0.0003	0.0007	0.4772	0.6642	NEGATIVE
Eggerthella	0.0006	0.0007	0.4854	0.6642	NEGATIVE
Coprobacter	0.0007	0.0012	0.4775	0.6642	NEGATIVE

Genus	Mean Relative Abundance Bacteremia Group	Mean Relative Abundance No Bacteremia Group	p-value	Adjusted p-value	Association Direction
Phascolarctobacterium	0.0049	0.0061	0.4809	0.6642	NEGATIVE
Varibaculum	7.3E-06	2.2E-06	0.4941	0.6666	POSITIVE
Lysinibacillus	1.9E-05	0.0001	0.5075	0.6806	NEGATIVE
Lachnospiraceae/Fusobacteriaceae	0.0009	0.0012	0.5115	0.6820	NEGATIVE
Hafnia	0.0002	0.0003	0.5282	0.7001	NEGATIVE
Lactobacillus/Lactiplantibacillus	0.0016	0.0010	0.5359	0.7062	POSITIVE
Akkermansia	0.0158	0.0123	0.5502	0.7210	POSITIVE
Gemella	0.0006	0.0009	0.5535	0.7211	NEGATIVE
Dialister	0.0014	0.0011	0.5650	0.7320	POSITIVE
Pantoea/Leclercia	0.0002	0.0001	0.5693	0.7333	POSITIVE
Catenibacterium	0.0005	0.0002	0.5815	0.7406	POSITIVE
Finegoldia	0.0016	0.0011	0.5808	0.7406	POSITIVE
Prevotellamassilia	0.0021	0.0013	0.6075	0.7695	POSITIVE
Phocea	0.0001	0.0001	0.6164	0.7765	POSITIVE
Lactonifactor	3.0E-05	1.7E-05	0.6217	0.7788	POSITIVE
Bhargavaea	6.9E-06	1.4E-05	0.6421	0.7793	NEGATIVE
Howardella	0.0001	0.0001	0.6466	0.7793	POSITIVE
Scardovia	0.0003	0.0004	0.6506	0.7793	NEGATIVE
Sutterella	0.0004	0.0003	0.6406	0.7793	POSITIVE
Bilophila	0.0007	0.0006	0.6415	0.7793	POSITIVE
Rothia	0.0007	0.0010	0.6491	0.7793	NEGATIVE
Megasphaera	0.0017	0.0022	0.6528	0.7793	NEGATIVE
Neglecta	0.0024	0.0029	0.6480	0.7793	NEGATIVE
Prevotella	0.0075	0.0059	0.6278	0.7793	POSITIVE
Leptotrichia	0.0005	0.0008	0.6631	0.7874	NEGATIVE
Eubacterium/Mediterraneibacter	1.3E-06	6.5E-07	0.6696	0.7905	POSITIVE
Dysosmobacter/Oscillibacter	0.0001	0.0001	0.6726	0.7905	NEGATIVE
Ihubacter	0.0001	0.0001	0.6886	0.8051	POSITIVE
Cuneatibacter	0.0001	0.0001	0.6922	0.8053	POSITIVE
Merdibacter	0.0002	0.0002	0.7296	0.8444	NEGATIVE
Pseudoruminococcus	0.0003	0.0003	0.7534	0.8632	NEGATIVE
Longicatena	0.0011	0.0014	0.7514	0.8632	NEGATIVE
Anaerofilum	2.7E-05	3.3E-05	0.7658	0.8687	NEGATIVE
Obesumbacterium	0.0001	0.0001	0.7633	0.8687	NEGATIVE
Desulfovibrio	0.0003	0.0002	0.7737	0.8733	POSITIVE
Haemophilus	0.0002	0.0002	0.7812	0.8765	NEGATIVE
Eubacterium	0.0028	0.0030	0.7843	0.8765	NEGATIVE
Peptococcus	0.0001	0.0002	0.7998	0.8767	NEGATIVE
Bifidobacterium	0.0094	0.0103	0.7956	0.8767	NEGATIVE
Erysipelatoclostridium	0.0214	0.0226	0.7963	0.8767	NEGATIVE
Klebsiella	0.0259	0.0281	0.7980	0.8767	NEGATIVE
Pseudoflavonifractor	0.0002	0.0002	0.8049	0.8781	POSITIVE
Lachnoanaerobaculum	0.0002	0.0001	0.8157	0.8856	POSITIVE
Pseudoflavonifractor/Clostridium	0.0004	0.0004	0.8230	0.8893	NEGATIVE
Faecalimonas	0.0034	0.0031	0.8385	0.9018	POSITIVE
Clostridium	0.0075	0.0071	0.8528	0.9129	POSITIVE
Lactococcus	0.0024	0.0022	0.8704	0.9274	POSITIVE
Robinsoniella	5.0E-05	4.4E-05	0.8894	0.9432	POSITIVE
Enterobacter	0.0103	0.0109	0.8953	0.9451	NEGATIVE
Clostridiales Family XIII	0.0004	0.0004	0.9095	0.9556	NEGATIVE
Desulfovibrionaceae	2.1E-05	2.4E-05	0.9153	0.9573	NEGATIVE
Hungateiclostridiaceae	0.0002	0.0002	0.9257	0.9594	POSITIVE
Eubacteriaceae	0.0005	0.0005	0.9231	0.9594	POSITIVE
Murimonas	1.8E-05	1.7E-05	0.9358	0.9611	POSITIVE
Fusobacterium	0.0019	0.0018	0.9320	0.9611	POSITIVE
Massilimicrobiota	0.0005	0.0005	0.9478	0.9690	NEGATIVE

Genus	Mean Relative Abundance Bacteremia Group	Mean Relative Abundance No Bacteremia Group	p-value	Adjusted p-value	Association Direction
Catenibacillus	3.2E-05	3.3E-05	0.9718	0.9891	NEGATIVE
Massilioclostridium	8.6E-06	8.4E-06	0.9877	0.9966	POSITIVE
Veillonella	0.0077	0.0077	0.9879	0.9966	POSITIVE
Ellagibacter	3.7E-05	3.7E-05	0.9951	0.9995	NEGATIVE
Amedibacterium	0.0001	0.0001	1.0000	1.0000	POSITIVE
