# Supplementary material for: Gut Microbiota and Intestinal Monodomination as a Predictor for Bacteremia in Allogeneic Hematopoietic Cell Transplant Recipients
Source: J Infect Dis. 2026 Feb 24;234(1):e81–9. doi: 10.1093/infdis/jiag005 (PMC13431778; doi:10.1093/infdis/jiag005)
Supplement: jiag005_Supplementary_Data [file jiag005_supplementary_data.zip › Supplementary_Table_05.pdf]

Supplementary Table 5. Organism Match Between Stool Sample and Blood Culture

	Bacteremia events (n = 114)
Species Match	67 (58.8%)
Genus Match	13 (11.4%)
No Match	34 (29.8%)
