# Supplementary material for: Gut Microbiota and Intestinal Monodomination as a Predictor for Bacteremia in Allogeneic Hematopoietic Cell Transplant Recipients
Source: J Infect Dis. 2026 Feb 24;234(1):e81–9. doi: 10.1093/infdis/jiag005 (PMC13431778; doi:10.1093/infdis/jiag005)
Supplement: jiag005_Supplementary_Data [file jiag005_supplementary_data.zip › Supplementary_Table_06.pdf]

Supplementary Table 6. Comparing PPV Utilizing Pre- and Post-Bacteremia Stool Samples v. Exclusively Pre-Bacteremia Stool Samples. We repeated our PPV analysis comparing two windows for sampling in relation to bacteremia event. The left side represents ± 30 days from the bacteremia event (as seen in Figure 4), while the right side represents a window limited to the 30 days preceding the event. TP = true positives, FN = false negatives, FP = false positives, TN = true negatives, PPV = positive predictive value (highlighted in blue), NPV = negative predictive value.

		Plus/Minus 30 Days								Minus 30 Days							
Organism	Threshold	TP	FN	FP	TN	Sensitivity	Specificity	PPV	NPV	TP	FN	FP	TN	Sensitivity	Specificity	PPV	NPV
Viridans streptococci	0	16	0	234	23	1.000	0.089	0.064	1.000	14	2	234	23	0.875	0.089	0.056	0.920
	0.1	7	9	65	192	0.438	0.747	0.097	0.955	5	11	65	192	0.313	0.747	0.071	0.946
	0.3	1	15	22	235	0.063	0.914	0.043	0.940	1	15	22	235	0.063	0.914	0.043	0.940
	0.5	1	15	13	244	0.063	0.949	0.071	0.942	1	15	13	244	0.063	0.949	0.071	0.942
E. coli	0	7	4	134	128	0.636	0.489	0.050	0.970	7	4	134	128	0.636	0.489	0.050	0.970
	0.1	6	5	91	171	0.545	0.653	0.062	0.972	6	5	91	171	0.545	0.653	0.062	0.972
	0.3	6	5	65	197	0.545	0.752	0.085	0.975	5	6	65	197	0.455	0.752	0.071	0.970
	0.5	3	8	42	220	0.273	0.840	0.067	0.965	3	8	42	220	0.273	0.840	0.067	0.965
Klebsiella	0	6	3	145	119	0.667	0.451	0.040	0.975	6	3	145	119	0.667	0.451	0.040	0.975
	0.1	5	4	81	183	0.556	0.693	0.058	0.979	5	4	81	183	0.556	0.693	0.058	0.979
	0.3	3	6	46	218	0.333	0.826	0.061	0.973	3	6	46	218	0.333	0.826	0.061	0.973
	0.5	2	7	26	238	0.222	0.902	0.071	0.971	2	7	26	238	0.222	0.902	0.071	0.971
Coag-negative *Staphylococcus*	0	24	18	103	128	0.571	0.554	0.189	0.877	13	29	103	128	0.310	0.554	0.112	0.815
	0.1	8	34	24	207	0.190	0.896	0.250	0.859	3	39	24	207	0.071	0.896	0.111	0.841
	0.3	7	35	13	218	0.167	0.944	0.350	0.862	3	39	13	218	0.071	0.944	0.188	0.848
	0.5	6	36	10	221	0.143	0.957	0.375	0.860	2	40	10	221	0.048	0.957	0.167	0.847
Enterococcus	0	6	0	211	56	1.000	0.210	0.028	1.000	6	0	211	56	1.000	0.210	0.028	1.000
	0.1	4	2	70	197	0.667	0.738	0.054	0.990	4	2	70	197	0.667	0.738	0.054	0.990
	0.3	4	2	43	224	0.667	0.839	0.085	0.991	4	2	43	224	0.667	0.839	0.085	0.991
	0.5	4	2	36	231	0.667	0.865	0.100	0.991	3	3	36	231	0.500	0.865	0.077	0.987
Gemella	0	2	4	66	201	0.333	0.753	0.029	0.980	1	5	66	201	0.167	0.753	0.015	0.976
	0.1	0	6	4	263	0.000	0.985	0.000	0.978	0	6	4	263	0.000	0.985	0.000	0.978
	0.3	0	6	1	266	0.000	0.996	0.000	0.978	0	6	1	266	0.000	0.996	0.000	0.978
	0.5	0	6	0	267	0.000	1.000	NA	0.978	0	6	0	267	0.000	1.000	NA	0.978
Staphylococcus aureus	0	2	3	2	266	0.400	0.993	0.500	0.989	1	4	2	266	0.200	0.993	0.333	0.985
	0.1	0	5	0	268	0.000	1.000	NA	0.982	0	5	0	268	0.000	1.000	NA	0.982
	0.3	0	5	0	268	0.000	1.000	NA	0.982	0	5	0	268	0.000	1.000	NA	0.982
	0.5	0	5	0	268	0.000	1.000	NA	0.982	0	5	0	268	0.000	1.000	NA	0.982
