# Supplementary material for: Gut Microbiota and Intestinal Monodomination as a Predictor for Bacteremia in Allogeneic Hematopoietic Cell Transplant Recipients
Source: J Infect Dis. 2026 Feb 24;234(1):e81–9. doi: 10.1093/infdis/jiag005 (PMC13431778; doi:10.1093/infdis/jiag005)
Supplement: jiag005_Supplementary_Data [file jiag005_supplementary_data.zip › Supplementary_Table_07.pdf]

Supplementary Table 7. PPV of Bacteremia Among Patients with and without Gut GvHD. Utilizing the same methods as Figure 4, we performed additional analyses to calculate the PPV for each of the top 7 bacteremia organisms among patients with any level of gut GvHD (left) and patients without gut GvHD (right). Several of the generated PPVs are based on very low sampling numbers. TP = true positives, FN = false negatives, FP = false positives, TN = true negatives, PPV = positive predictive value

		Participants w/ Gut GvHD			Participants w/no Gut GvHD		
**Organism**	**threshold**	**TP**	**FP**	**PPV**	**TP**	**FP**	**PPV**
Viridans streptococci	0	15	131	0.1027	1	105	0.0094
	0.1	7	42	0.1429	0	24	0.0000
	0.3	1	18	0.0526	0	4	0.0000
	0.5	1	12	0.0769	0	1	0.0000
E. coli	0	3	83	0.0349	4	51	0.0727
	0.1	2	57	0.0339	4	34	0.1053
	0.3	2	37	0.0513	4	28	0.1250
	0.5	1	22	0.0435	2	20	0.0909
Klebsiella	0	6	90	0.0625	0	55	0.0000
	0.1	5	50	0.0909	0	31	0.0000
	0.3	3	28	0.0968	0	18	0.0000
	0.5	2	14	0.1250	0	12	0.0000
**Coag-negative *Staphylococcus***	0	15	66	0.1852	9	37	0.1957
	0.1	4	14	0.2222	4	10	0.2857
	0.3	4	9	0.3077	3	4	0.4286
	0.5	4	9	0.3077	2	1	0.6667
Enterococcus	0	5	123	0.0391	1	90	0.0110
	0.1	4	38	0.0952	0	32	0.0000
	0.3	4	22	0.1538	0	21	0.0000
	0.5	4	18	0.1818	0	18	0.0000
Gemella	0	1	38	0.0256	1	28	0.0345
	0.1	0	3	0.0000	0	1	0.0000
	0.3	0	1	0.0000	0	0	NA
	0.5	0	0	NA	0	0	NA
Staphylococcus aureus	0	1	2	0.3333	1	0	1.0000
	0.1	0	0	NA	0	0	NA
	0.3	0	0	NA	0	0	NA
	0.5	0	0	NA	0	0	NA
