# Supplementary material for: Gut Microbiota and Intestinal Monodomination as a Predictor for Bacteremia in Allogeneic Hematopoietic Cell Transplant Recipients
Source: J Infect Dis. 2026 Feb 24;234(1):e81–9. doi: 10.1093/infdis/jiag005 (PMC13431778; doi:10.1093/infdis/jiag005)
Supplement: jiag005_Supplementary_Data [file jiag005_supplementary_data.zip › Supplementary_Table_08.pdf]

Supplementary Table 8. PPV of Bacteremia Among Patients with Early and Late CoNS Bacteremia. Utilizing the same methods as Figure 4, we performed additional analyses to calculate the PPV among patients with early and late CoNS bacteremia events. CoNS bacteremia events were split into two time groups using the median of all CoNS events. Early refers to Day 35 and prior and late refers to any time after day 35. The Threshold column represents the relative abundance cut-off that was used to generate the PPV. TP = true positives, FN = false negatives, FP = false positives, TN = true negatives, PPV = positive predictive value (Highlighted in blue), NPV = negative predictive value.

Time Group	Threshold	TP	FN	FP	TN	Sensitivity	Specificity	PPV	NPV
Early	0	19	5	103	128	0.7917	0.5541	0.1557	0.9624
Late	0	5	7	103	128	0.4167	0.5541	0.0463	0.9481
Early	0.1	7	17	24	207	0.2917	0.8961	0.2258	0.9241
Late	0.1	1	11	24	207	0.0833	0.8961	0.0400	0.9495
Early	0.3	6	18	13	218	0.2500	0.9437	0.3158	0.9237
Late	0.3	1	11	13	218	0.0833	0.9437	0.0714	0.9520
Early	0.5	5	19	10	221	0.2083	0.9567	0.3333	0.9208
Late	0.5	1	11	10	221	0.0833	0.9567	0.0909	0.9526
